# Engineering plant tandem kinase immune receptors expands effector recognition profiles

**DOI:** 10.1126/sciadv.aef2426

**Published:** 2026-09-23

**Authors:** Daniel S. Yu, Rafał Zdrzałek, Emi Katayama, Hitomi Akiyama, Lucy Daykin, Nathan J. Williams, Iwan Goodridge, Soichiro Asuke, Mark J. Banfield

**Affiliations:** ^1^ Department of Biochemistry and Metabolism, John Innes Centre, Norwich Research Park, Norwich, NR4 7UH, UK.; ^2^ Graduate School of Agricultural Science, Kobe University, Kobe 657-8501, Japan.

## Abstract

Plant intracellular immune receptors are widely deployed in breeding to protect crops from disease. In addition to nucleotide-binding leucine-rich repeat receptors (NLRs), tandem kinase proteins (TKPs) have recently emerged as an important family of immune receptors within staple cereal food crops, but how TKPs recognize effectors and whether they are amenable to engineering is essentially unknown. Here, we show that the barley and wheat TKPs Rmo2 and Rwt7 recognize different blast fungus effectors via their integrated HMA domains using different protein interfaces with nanomolar binding affinity. Structural analysis pinpointed interface residues that dictate effector recognition and enabled engineering of dual-specificity TKPs. These results establish integrated HMA domains as programmable modules within TKPs for designing new specificities in plant immunity for diseases relevant to global agriculture.

## INTRODUCTION

Globally, 20–30% of cereal crop production (including wheat, rice and barley) is lost each year to plant diseases (*1*–*3*). Plant disease defenses are triggered by cell surface or intracellular receptors that recognize pathogen-associated molecular patterns (PAMPs) or effectors released from pathogens during infection (*4*). Structural characterization of intracellular nucleotide-binding leucine-rich repeat receptors (NLRs) (*5*) has elucidated the activation mechanisms for these plant immune receptors, highlighting previously unrecognized potential for engineering disease resistance (*6*–*10*).

Despite the importance of NLRs, up to 35% of race-specific resistance genes from wheat and barley do not encode canonical NLRs (*11*). Genes encoding kinase-fusion proteins (KFPs) have emerged as a major source of resistance within cereal crops for multiple agriculturally relevant diseases (*12*–*24*). Most cloned KFPs are tandem kinase proteins (TKPs), with some of the best characterized examples being Rpg1, Sr62 and RWT4 (*12*, *25*–*27*). While Sr62 and RWT4 are both TKPs, they recognize effectors via different mechanisms. The first kinase domain of Sr62 has a variable beta-finger domain predicted to dimerize and facilitate the binding of AvrSr62 (*25*). RWT4 is predicted to bind AvrPWT4 via its N-terminal kinase duplication (KDup) domain and pseudokinase domain, and substitutions within the KDup domain that prevent effector binding result in disease susceptibility (*27*).

Plant receptors have evolved non-canonical integrated domains that function as effector decoys. For example, a subset of NLRs, such as Pik-1 and RGA5 in rice, have an integrated heavy metal-associated (HMA) domain that mimics the effector host target that facilitates effector binding and immune activation (*28*, *29*). Remarkably, integrated HMA domains are also found in TKPs. Rpg1 has an HMA domain at its N terminus that went unnoticed until recent advancements in structural modeling (*30*). Furthermore, a strikingly similar domain structure containing an N-terminal HMA domain was discovered in the TKPs Rmo2 (of barley) and Rwt7 (of wheat), which recognize the effectors PBY2 and PWT7, respectively, from different pathotypes of the blast fungus *Magnaporthe oryzae* (*24*). Whether these domains function to recognize effectors and promote immune responses is unknown.

In this study, we investigated the role of the integrated HMA domains of the TKP orthologues Rmo2 and Rwt7 in effector recognition and initiation of immune signaling. We showed the integrated HMA domains in TKPs are direct effector-binding modules by elucidating the structural and biochemical basis of effector binding and immune signaling specificity. Furthermore, based on the structures, we engineered Rmo2 and Rwt7 with expanded responses that include previously unrecognized effectors. Our work demonstrates that TKP integrated domain engineering is a viable approach to develop TKP receptors with expanded recognition profiles.

## RESULTS

### TKP integrated HMA domains directly interact with pathogen effectors in vitro

**Fig. 1.**
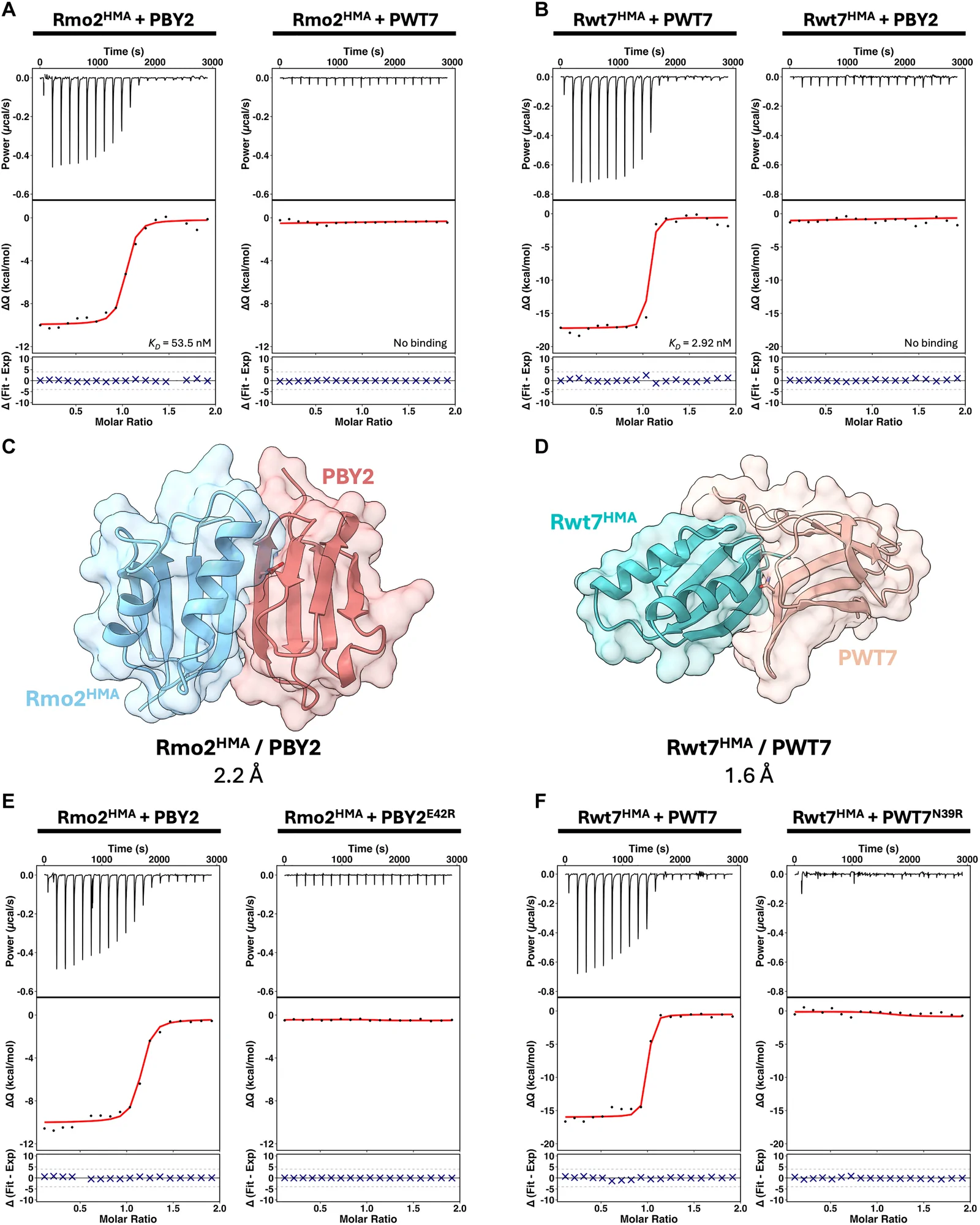
Biophysical and structural analysis of binding specificities from the integrated HMA domains of TKPs. (**A**) Isothermal titration calorimetry (ITC) assay of Rmo2^HMA^ mixed with PBY2 or PWT7, and of (**B**) Rwt7^HMA^ mixed with PWT7 or PBY2. (**C**) Crystal structures of the Rmo2^HMA^/PBY2 (PDB: 9TFO) and (**D**) Rwt7^HMA^/PWT7 (PDB: 9TFP) complexes. Key residues within the effectors involved in interacting with the HMA domains (E42 from PBY2 and N39 from PWT7) are shown as sticks. (**E**) ITC assay of Rmo2^HMA^ mixed with PBY2 or PBY2^E42R^, and of (**F**) Rwt7^HMA^ mixed with PWT7 or PWT7^N39R^. All ITC experiments were performed in triplicate and were analyzed using AFFINImeter.

Integrated HMA domains in plant NLRs recognize specific effectors, thereby initiating immune responses (*28*, *29*). Similarly, HMA-containing TKP receptor orthologues Rmo2 and Rwt7 recognize different *M. oryzae* effectors. Barley lines harboring *Rmo2* are resistant to *M. oryzae* pathotypes expressing *PBY2* but not *PWT7*, while wheat lines carrying *Rwt7* are resistant to *M. oryzae* pathotypes expressing *PWT7* but not *PBY2* (*24*). The orthologous TKP receptors Rmo2, Rwt7, Rpg1, WTK4 and G012400 are highly variable in sequence across their HMA domains (38–62% HMA identity between Rmo2, Rwt7, Rpg1 and WTK4, 82% identity between Rpg1 and G012400), while their pseudokinase and kinase domain sequences are more similar (74–83% pseudokinase domain identity, 67–81% kinase domain identity) (fig. S1). Therefore, we hypothesized that the integrated HMA domains of Rmo2 and Rwt7 function as bait domains, similar to those found in NLR receptors. To test this, we determined the binding specificities and affinities of the integrated HMA domains of Rmo2 and Rwt7 towards PBY2 and PWT7 in vitro. We purified recombinant HMA domains (Rmo2^HMA^ and Rwt7^HMA^) and effectors (PBY2 and PWT7) and performed analytical size exclusion chromatography (aSEC). We observed shifts in peak elution time when Rmo2^HMA^ and PBY2, or Rwt7^HMA^ and PWT7 were pre-incubated together, relative to the elution times of the individual proteins, indicating direct interaction. We detected no peak shift when the respective HMA domains were mixed with their non-corresponding effectors (fig. S2). Quantitative analysis of the binding affinities using isothermal titration calorimetry (ITC) also demonstrated the specificity of the interaction between Rmo2^HMA^ and PBY2, and between Rwt7^HMA^ and PWT7 (Fig. 1, A and B). These interactions displayed nanomolar binding affinities, with *K_D_* values of 53.5 nM for Rmo2^HMA^ with PBY2 and 2.92 nM for Rwt7^HMA^ with PWT7. Consistent with the aSEC experiments, there was no binding observed between each HMA domain and the non-corresponding effector in ITC experiments. Collectively, these data demonstrate that the HMA domains of Rmo2 and Rwt7 directly bind their corresponding effectors with high affinity and dictate recognition specificity for these receptors.

### Structure-informed interface mutagenesis disrupts protein interactions in vitro

To understand the structural basis of the interactions of Rmo2^HMA^ with PBY2 and of Rwt7^HMA^ with PWT7, we determined the structures of their complexes with *X*-ray crystallography. Accordingly, we co-purified and obtained crystals for Rmo2^HMA^/PBY2 and Rwt7^HMA^/PWT7 HMA domain/effector pairs (fig. S3). We solved the crystal structures of Rmo2^HMA^/PBY2 and Rwt7^HMA^/PWT7 by molecular replacement using AlphaFold2 models as templates (*31*, *32*), reaching refined resolutions of 2.2 Å and 1.6 Å, respectively (Fig. 1, C and D, table S1). The effectors bound to spatially distinct interfaces on the HMA domains. The interface between Rmo2^HMA^ and PBY2 was predominantly mediated by polar main-chain interactions between the β3 of Rmo2^HMA^ and β2 of PBY2 with only limited polar side-chain interactions (D16 and K19 from Rmo2^HMA^ and E42 and S48 from PBY2) (fig. S4A). By contrast, the Rwt7^HMA^/PWT7 interface displayed many polar side-chain interactions across two faces of Rwt7^HMA^. Central to the interaction interface were the sidechains of N39 and D40 from PWT7, while the first residue of Rwt7^HMA^ (M1) formed extensive interactions with the effector (fig. S4, B and C). We tested whether the interaction interfaces observed in the structures underpin protein–protein interactions by introducing structure-informed single amino-acid substitutions in PBY2 (E42R) and PWT7 (N39R) to disrupt interactions with their respective HMA domains. We tested the binding of each recombinant effector mutant to its corresponding HMA domain by aSEC and ITC. No peak shift was observed by aSEC when PBY2^E42R^ and PWT7^N39R^ were incubated with Rmo2^HMA^ or Rwt7^HMA^, respectively (fig. S5). Similarly, each effector mutant showed no quantitative binding to its respective HMA domain by ITC (Fig. 1, E and F). Therefore, structure-informed mutagenesis of the effectors successfully disrupted the interactions with the respective HMA domains in vitro.

### Rmo2 and Rwt7 integrated HMA domains are baits for effectors in planta

**Fig. 2.**
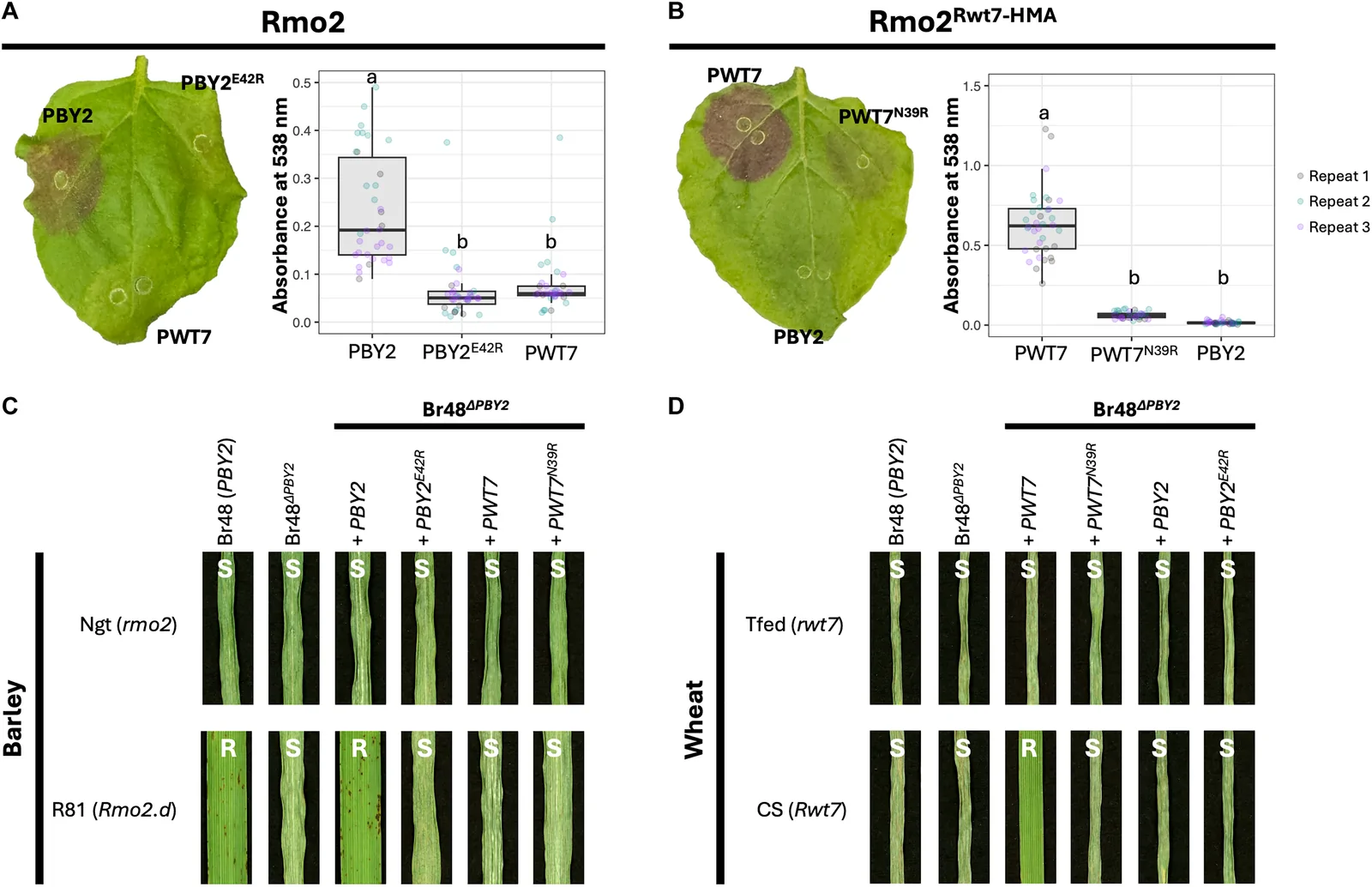
Structure-informed effector mutants no longer bind to or recognize their corresponding TKP *in planta*. (**A** and **B**) Split GAL4 RUBY assay showing betalain production in *Nicotiana benthamiana* leaves following co-expression of (A) *Rmo2-GAL4* (encoding Rmo2 with the DNA-binding domain of yeast GAL4 in its C terminus) with *PBY2-VP16*, *PBY2^E42R^-VP16*, or *PWT7-VP16* (encoding the effectors fused to the activation domain of VP16 at their C termini) or (B) *Rmo2^Rwt7-HMA^-GAL4* with *PWT7-VP16*, *PWT7^N39R^-VP16*, or *PBY2-VP16*. All expression cassettes were under the control of the cauliflower mosaic virus (CaMV) 35S promoter. Leaves were harvested 4 days after infiltration and chlorophyll was cleared with ethanol. Betalain was extracted from each infiltrated spot and absorbance was measured at 538 nm. Each experiment consisted of 6 or 12 leaf replicates, performed three times independently. Different letters indicate significant differences, as determined by one-way ANOVA and *post-hoc* Tukey’s honestly significant difference tests at *P <* 0.05. (**C** and **D**) Infection assay with *Magnaporthe oryzae* BR48 derived and complementation isolates on the barley cultivars ‘Nigrate’ (Ngt) and ‘Russia 81’ (R81), or (D) on the wheat cultivars ‘Transfed’ (Tfed) and ‘Chinese Spring’ (CS). Primary leaves of eight-day-old seedlings were spray-inoculated with *M. oryzae* isolates. Leaves were imaged 4 days post inoculation. Resistant (R) or susceptible (S) phenotypes are labeled.

To establish whether the in vitro interactions between HMA domains and effectors reflect effector binding to full-length receptors in plant cells, we used the split GAL4 RUBY assay (*33*). Areas of *Nicotiana benthamiana* leaves co-expressing *Rmo2-GAL4* (encoding Rmo2 fused with the DNA-binding domain of yeast GAL4) and *PBY2-VP16* (encoding PBY2 fused with VP16) accumulated red pigments due to transcriptional activation of betalain production, indicative of protein–protein interactions in this assay (Fig. 2A). Co-expression of *Rmo2-GAL4* and *PWT7-VP16* did not lead to significant betalain accumulation, confirming the specificity of the effector interactions. Furthermore, leaves co-expressing *Rmo2-GAL4* with the effector mutant *PBY2^E42R^-VP16* did not accumulate betalain, showing that the E42R mutation in PBY2 disrupted interaction with Rmo2 in plant cells. Accumulation of proteins was confirmed in plant tissues by immunoblot (fig. S7). We attempted the split GAL4 RUBY assay with Rwt7-GAL4, but detected little betalain production for any combination, suggesting that Rwt7 may not be suitable for this assay (fig. S6). As replacing the HMA domain of Rmo2 with Rwt7^HMA^ switches recognition specificity of Rmo2 from PBY2 to PWT7 (*24*), we generated the *Rmo2^Rwt7-HMA^-GAL4* construct and co-expressed it with *PWT7-VP16*, *PBY2-VP16*, or *PWT7^N39R^-VP16* (Fig. 2B). We observed significantly higher betalain production in the presence of PWT7 than with PBY2 or PWT7^N39R^, suggesting that the chimeric receptor interacts with PWT7 instead of PBY2, and that the N39R mutation in PWT7 disrupts interaction with Rmo2^Rwt7-HMA^.

### The integrated HMA domains of Rmo2 and Rwt7 bind effectors for immunity in cereals

To link direct receptor–effector interactions to susceptibility or resistance phenotypes during infection, we performed spray inoculation assays on barley and wheat cultivars, challenging them with *M. oryzae* strains expressing effectors or effector mutants (Fig. 2, C and D). The barley cultivar ‘Nigrate’ (Ngt, lacking *Rmo2*) was susceptible to *M. oryzae* strain Br48, which harbors *PBY2*, but the barley cultivar ‘Russia 81’ (R81, carrying *Rmo2*) was resistant (Fig. 2C). The *PBY2* deletion strain (Br48^ΔPBY2^) causes disease on R81 (*24*). Indeed, R81 challenged with Br48^ΔPBY2^ displayed susceptibility, as did R81 challenged with Br48^ΔPBY2^ complemented with *PBY2^E42R^* (Fig. 2C). R81 was resistant to Br48^ΔPBY2^ complemented with *PBY2*. The Ngt and R81 cultivars were both susceptible to Br48^ΔPBY2^ complemented with *PWT7* or *PWT7^N39R^*. The wheat cultivars ‘Transfed’ (Tfed, lacking *Rwt7*) and ‘Chinese Spring’ (CS, containing *Rwt7*) were both susceptible to infection with Br48^ΔPBY2^ (lacking *PWT7*) (Fig. 2D). CS was resistant to Br48^ΔPBY2^ complemented with *PWT7*, but susceptible to Br48^ΔPBY2^ complemented with *PWT7^N39R^*. Tfed and CS were both susceptible to Br48^ΔPBY2^ complemented with *PBY2* or *PBY2^E42R^*. Susceptibility and resistance phenotypes were quantified for the two independent experiments (fig. S8).

We conclude that disrupting the interaction between effectors and their corresponding receptor HMA domains prevents recognition by Rmo2 and Rwt7, confirming the critical role of the HMA domain as an integrated effector bait domain within these receptors.

### Structure-informed resurfacing of TKP integrated HMA domains expands their binding profiles

Rmo2^HMA^ and Rwt7^HMA^ specifically interact with PBY2 and PWT7, respectively (Fig. 1, A and B). In the crystal structures, the HMA domains of Rmo2 and Rwt7 interacted with their coresponding effectors via unique, spatially distinct interfaces (fig. S9). We thus wondered if resurfacing of the Rmo2 and Rwt7 integrated HMA domains could switch effector specificity and generate a receptor that could bind and respond to both PBY2 and PWT7.

**Fig. 3.**
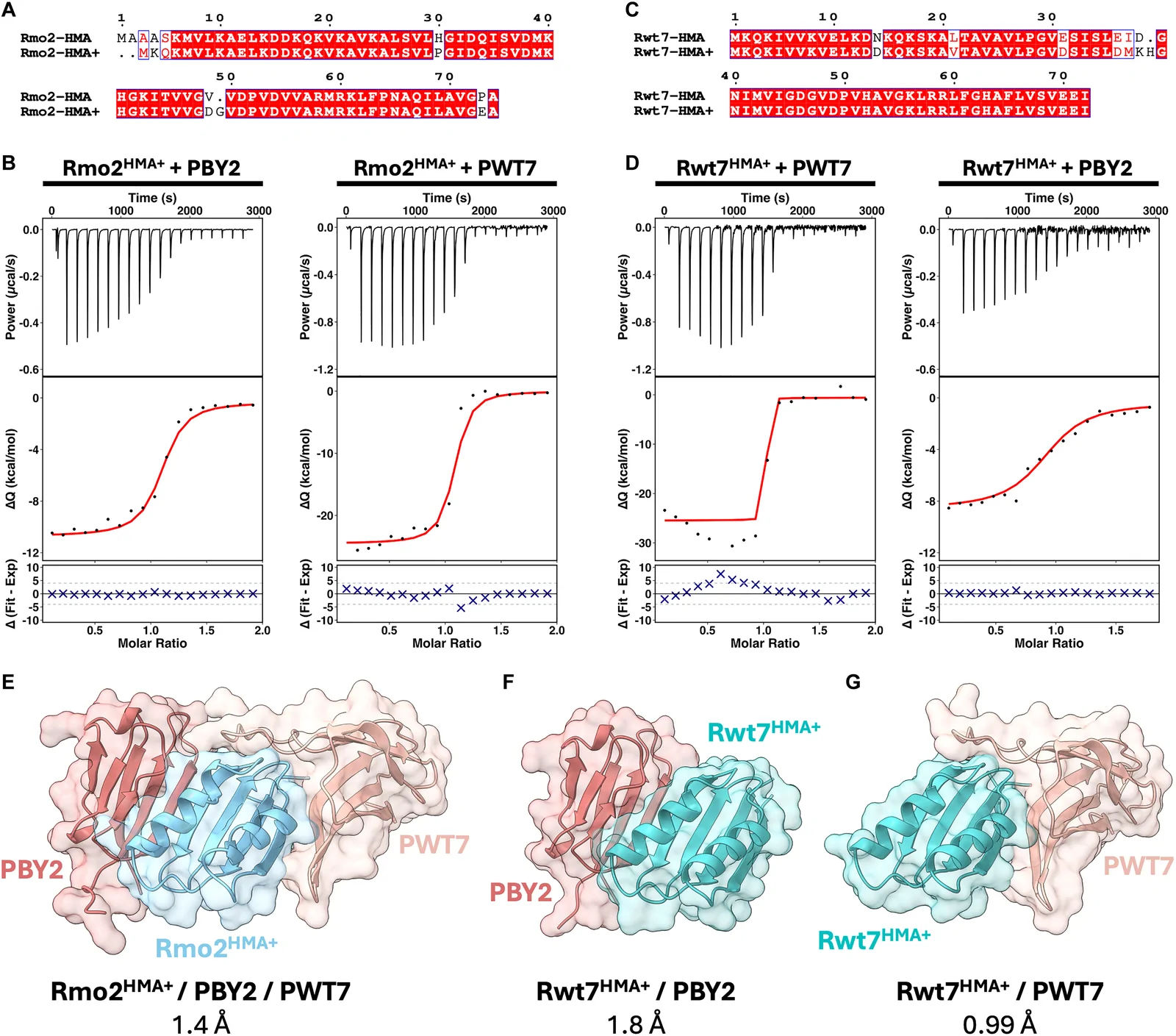
Resurfacing of the integrated HMA domains from TKPs confers binding to non-corresponding effectors in vitro. (**A**) Amino-acid sequence alignment of Rmo2^HMA^ and the resurfaced Rmo2^HMA+^. (**B**) Binding of Rmo2^HMA+^ to PBY2 or PWT7 by ITC. (**C**) Amino-acid sequence alignment of Rwt7^HMA^ and the resurfaced Rwt7^HMA+^. (**D**) ITC analysis of Rwt7^HMA+^ with PWT7 or PBY2. (**E** to **G**) Crystal structures of (E) the Rmo2^HMA+^/PBY2/PWT7 tripartite complex (PDB: 9TFQ), (F) the Rwt7^HMA+^/PBY2 (PDB: 9TFS) co-structure, and (G) the Rwt7^HMA+^/PWT7 (PDB: 9TFT) co-structure.

We resurfaced Rmo2^HMA^ along the Rwt7^HMA^/PWT7 interface, changing residues in Rmo2^HMA^ to those found in Rwt7^HMA^ (MAAAS^1–5^ to MKQ, H^30^ to P, V^49^ to DG, P^73^ to E), generating Rmo2^HMA+^ (Fig. 3A). ITC assays confirmed that engineered Rmo2^HMA+^ retains nanomolar binding affinity to PBY2, as did native Rmo2^HMA^ (Fig. 3B). Then we tested whether Rmo2^HMA+^ could bind to PWT7. In contrast to Rmo2^HMA^, which showed no binding to PWT7 (Fig. 1A), Rmo2^HMA+^ bound to PWT7 with nanomolar affinity (Fig. 3B). Similarly, we resurfaced Rwt7^HMA^ to generate Rwt7^HMA+^ (N^14^ to D, L^21^ to V, E^31^ to D, EID^36–38^ to DMKH) (Fig. 3C). Using ITC, we showed that engineered Rwt7^HMA+^ still binds to PWT7, but also gained binding to PBY2 with nanomolar affinity (Fig. 3D). We obtained crystals and determined the structures of Rwt7^HMA+^ co-purified with PBY2 or PWT7, and resolved a tripartite complex containing Rmo2^HMA+^, PBY2, and PWT7 (Fig. 3, E to G). The crystal structures of Rmo2^HMA+^ and Rwt7^HMA+^ in complex with their gain-of-binding interacting effectors were consistent with the native interactions (figs. S10 and S11 and table S1). That Rmo2^HMA+^ can bind two effectors raises the potential of engineering TKP immune receptors to respond to multiple effectors simultaneously.

**Fig. 4.**
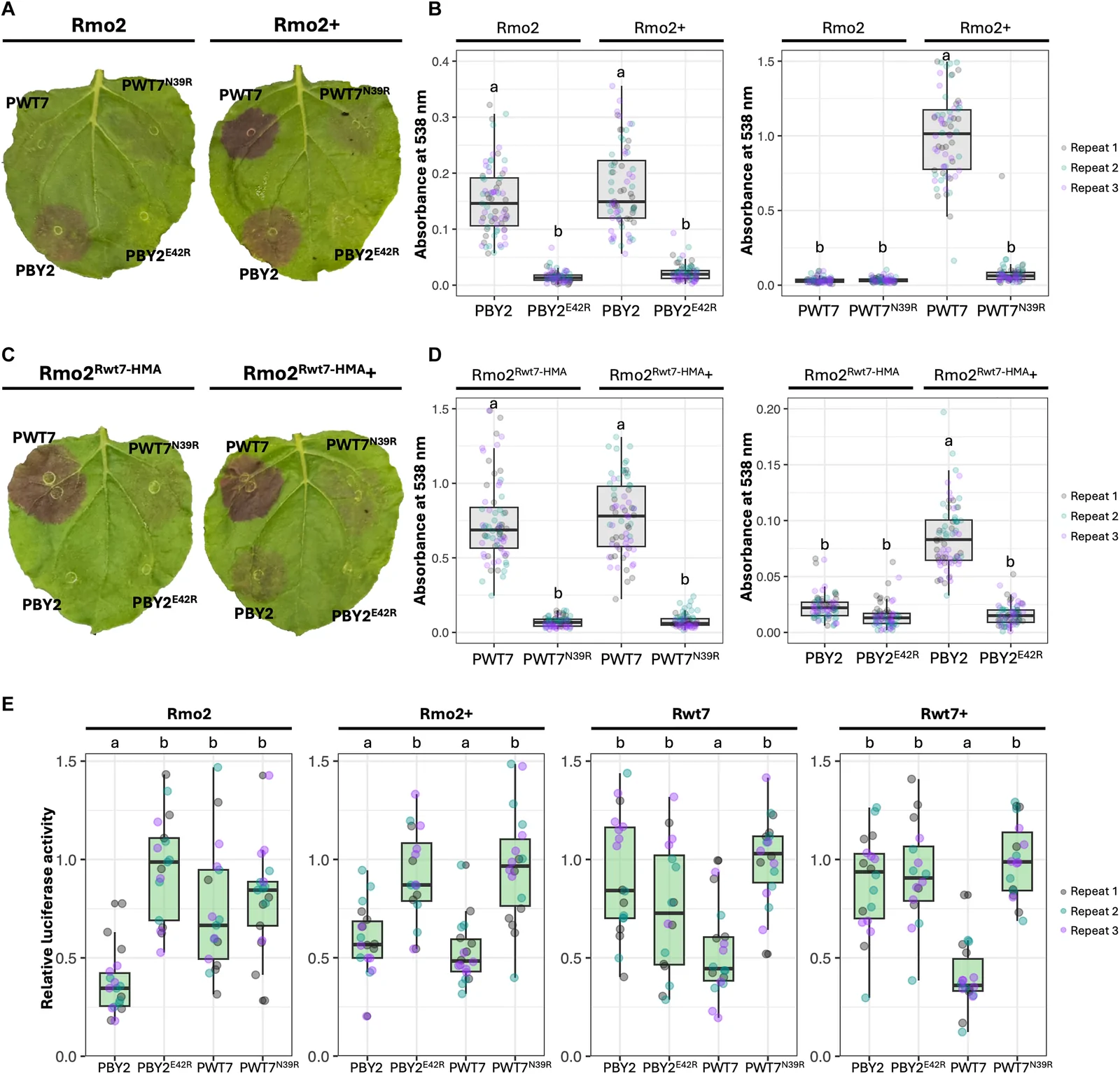
Incorporating the resurfaced HMA domains within TKPs expands their recognition profiles. (**A**) Split GAL4 RUBY assay showing betalain production in *N. benthamiana* leaves following co-expression of *Rmo2-GAL4* or *Rmo2 + −GAL4* with *PBY2-VP16*, *PBY2^E42R^-VP16*, *PWT7-VP16*, or *PWT7^N39R^-VP16*. (**B**) Quantification of betalain production from *N. benthamiana* leaves co-expressing *Rmo2-GAL4* or *Rmo2 + −GAL4* with PBY2-VP16, PBY2^E42R^-VP16, PWT7-VP16, or PWT7^N39R^-VP16. (**C**) Split GAL4 RUBY assay showing betalain production in *N. benthamiana* leaves following co-expression of *Rmo2^Rwt7-HMA^-GAL4* or *Rmo2^Rwt7-HMA^ + -GAL4* with *PBY2-VP16*, *PBY2^E42R^-VP16*, *PWT7-VP16*, or *PWT7^N39R^-VP16*. (**D**) Betalain quantification of *Rmo2^Rwt7-HMA^-GAL4* or *Rmo2^Rwt7-HMA^ + -GAL4* co-expressed with *PBY2-VP16*, *PBY2^E42R^-VP16*, *PWT7-VP16*, or *PWT7^N39R^-VP16*. Leaves were harvested and imaged 4 days after co-infiltration. Leaves were incubated in ethanol until all chlorophyll was cleared. Leaf discs were taken from each infiltration spot after chlorophyll clearing. Betalain was extracted in water and quantified by absorbance at 538 nm. Each experiment consisted of 24 leaf replicates, performed three times independently. Different letters indicate significant differences, as determined by one-way ANOVA and *post-hoc* Tukey’s honestly significant difference tests at *P* < 0.05. (**E**) Relative luciferase activity in wheat protoplasts (from the Kronos cultivar) transfected with constructs encoding Rmo2, Rmo2+, Rwt7, or Rwt7+ in the presence of the effector PBY2 or PWT7, or the effector mutant PBY2^E42R^ or PWT7^N39R^. Luciferase activity is derived from a *proZmUbi:LUC* reporter and was normalized to the values obtained with *PBY2^E42R^* co-expressed with *Rmo2* or *Rmo2*+, and those with *PWT7^N39R^* co-expressed with *Rwt7* or *Rwt7*+. Experiments consisting of six technical replicates (replicate reactions from the same batch of protoplasts) were performed three times independently. Different letters indicate significant differences, as determined by one-way ANOVA and post hoc Tukey’s honestly significant difference tests at *P* < 0.05.

We then tested whether gain of effector binding to Rmo2^HMA+^ or Rwt7^HMA+^ in vitro expanded the binding and response of full-length receptors in plant cells. We therefore replaced Rmo2^HMA^ with Rmo2^HMA+^ in Rmo2 to generate full-length engineered Rmo2+. Using the split GAL4 RUBY assay in *N. benthamiana*, overall betalain production was comparable when *Rmo2-GAL4* or *Rmo2 + −GAL4* was co-expressed with *PBY2-VP16*, suggesting that Rmo2+ still binds to PBY2 *in planta* (Fig. 4, A and B). Importantly, we observed strong betalain production when *Rmo2 + −GAL4* was co-expressed with *PWT7-VP16*, in contrast to little betalain accumulation when *Rmo2-GAL4* was co-expressed with *PWT7-VP16*. This finding suggests that Rmo2+ gained binding to PWT7 *in planta*. As Rwt7 is not suitable for the split GAL4 RUBY assay, we used chimeric Rmo2^Rwt7-HMA^ as backbone to replace Rwt7^HMA^ with Rwt7^HMA+^, generating the engineered receptor Rmo2^Rwt7-HMA^+. Overall betalain production in the split GAL4 RUBY assay with *Rmo2^Rwt7-HMA^ + -GAL4* co-expressed with *PWT7-VP16* was comparable to that with *Rmo2^Rwt7-HMA^*, suggesting that Rmo2^Rwt7-HMA^+ retains binding to PWT7. Co-expressing *Rmo2^Rwt7-HMA^ + -GAL4* with *PBY2-VP16* also resulted in significant levels of betalain, in contrast to *Rmo2^Rwt7-HMA^-GAL4* co-expressed with *PBY2-VP16* (Fig. 4, C and D). This result suggests that Rmo2^Rwt7-HMA^+ gained binding to PBY2 *in planta*. When either engineered receptor construct (*Rmo2 + −GAL4* or *Rmo2^Rwt7-HMA^ + -GAL4*) was co-expressed with the effector mutant *PBY2^E42R^-VP16* or *PWT7^N39R^-VP16*, we measured significantly lower betalain production, clearly establishing that pigment production is associated with direct effector interactions with the HMA domains of the receptors. Taken together, these data demonstrate that the engineered receptors have gained binding to a non-corresponding effector in plant cells.

### Engineered integrated HMA domains expand recognition profiles of TKP receptors in cereal cells

We co-expressed wildtype and engineered TKPs with corresponding effectors in *N. benthamiana*, however no cell death was observed suggesting a missing component is needed for immune execution (fig. S12). We therefore used a cereal protoplast cell-death assay (*34*) to test whether the gain of effector binding by engineered TKPs was accompanied by expanded recognition and immune responses. In this assay, a reporter construct harboring the firefly luciferase (*LUC*) reporter gene driven by the maize *Ubiquitin* promoter is co-transfected with plasmids encoding relevant receptor/effector combinations into protoplasts prepared from the wheat cultivar ‘Kronos’, which lacks *Rwt7*. Low relative luciferase activity reflects immune activation (cell death). We measured significantly lower relative luciferase activity when constructs encoding intact receptors were co-transfected with constructs encoding the corresponding effector (*Rmo2* with *PBY2*, *Rwt7* with *PWT7*), but not with constructs encoding effector mutants or mismatched effectors (Fig. 4E), consistent with the susceptibility/resistance phenotypes during infection assays (Fig. 2, C and D). The engineered Rmo2+ and Rwt7+ receptors displayed generally lower luciferase activity compared to wild-type (fig. S13). Nonetheless, protoplasts expressing engineered *Rmo2*+ with either *PBY2* or *PWT7* emitted significantly lower relative luciferase activity than those co-transfected with the non-interacting effector mutants PBY2^E42R^ and PWT7^N39R^ (Fig. 4E), supporting the notion that Rmo2+ gained recognition of PWT7 and retained recognition of PBY2. The engineered Rwt7+ showed significantly lower luciferase activity in the presence of PWT7 but not PBY2, suggesting that although Rwt7^HMA+^ gained binding to PBY2 in vitro and *in planta*, this interaction did not translate into a recognition response.

## DISCUSSION

Here, we demonstrate that the integrated HMA domains within the barley TKP Rmo2 and the wheat TKP Rwt7 act as bait domains to directly bind effectors from the blast pathogen and are necessary and sufficient for defining specificity. Structural analyses of the integrated HMA domain/effector complexes revealed interfaces between these proteins that can be altered by mutation, either to prevent effector binding and disease resistance or to gain novel immune signaling profiles (fig. S14).

Although effector-binding integrated domains are an established feature of NLR immune receptors, their presence in TKPs has only recently emerged, leaving their roles underexplored. Extensive genome mining across the plant kingdom indicated that over 56% of TKPs harbor integrated domains (*30*). Importantly, mutations within the integrated domains of TKPs can lead to loss of disease resistance (*19*, *27*), demonstrating their importance for protein function. Of the cloned TKPs with integrated HMA domains, the HMA sequences are more divergent compared to their more conserved kinase domains (fig. S1). This observation suggests that these integrated HMA domains are under selective pressure to diversify, potentially to evolve new effector-binding capabilities. Allelic variation is apparent, for example, within *Rpg1* and *Rmo2* across barley cultivars, as alleles of *Rpg1* (*12*, *35*) and *Rmo2* (*36*) encode proteins with variable HMA domains that may bind to different cereal pathogen effectors.

The sequence diversity within HMA-containing TKPs can be leveraged for receptor engineering. For example, replacing Rmo2^HMA^ with Rwt7^HMA^ within Rmo2 causes a switch in recognition specificity from PBY2 to PWT7 in transgenic barley lines (*24*). While Rmo2 was successfully engineered to recognize PWT7 in a cereal protoplast assay, the engineered Rwt7 failed to recognize PBY2, despite PBY2 interacting with this engineered HMA domain in vitro (Figs. 3D and 4E). A similar phenomenon was observed with engineered RGA5, which did not lead to resistance in transgenic rice against *M. oryzae* expressing *Avr-PikD*, despite in vitro binding of the engineered RGA5 HMA domain to Avr-PikD (*37*). It was observed that the binding affinity of Rwt7^HMA+^ with PBY2 was lower than that of Rwt7^HMA^ with PWT7 and Rmo2^HMA^ with PBY2 (Figs. 2, B and D and 3D). Rwt7 may have a higher binding affinity requirement for activation than Rmo2 and the lower binding affinity of PBY2 may be insufficient to activate Rwt7 + −mediated immune responses. Another possibility is that PBY2 binds to Rwt7+ in a way that cannot activate the receptor for downstream immune signaling. Elucidating the mechanism of immunity mediated by the HMA domains of TKPs will greatly improve our ability to precisely engineer these receptors in the future.

Many characterized intracellular immune receptors require protein partners to convert pathogen perception into responses. Coiled-coil-type NLRs (CNLs) can be singletons (detect effectors and directly promote cell-death), but many “sensor” CNLs (that perceive effectors) signal through “helper” CNLs encoded by paired genes or as part of genetic networks (*5*). Notably, specific TKPs appear to also require an NLR for immune signaling following effector perception. Sr62^TK^, WTK3, and Rwt4 require the same partner NLR, highlighting how NLRs can act as signaling hubs for multiple TKPs (*25*, *26*). Whether Rmo2 and Rwt7 require an NLR to execute immune signaling after effector perception is currently unknown. In Rpg1, the catalytic lysine residues in the pseudokinase (K152) and kinase (K461) domains are both required for disease resistance (*38*), and one susceptible *Rpg1* allele (WBDC323) carries mutations within the pseudokinase domain (I269F, S319RF), suggesting that the pseudokinase domain is important for downstream signaling (*35*), as observed for Sr62^TK^ and Rwt4 (*25*, *27*).

Plant immune receptor engineering is an emerging tool to help curb crop losses to plant disease in the future by expanding the recognition profiles of receptors and the generation of new-to-nature disease resistance (*39*–*41*). Engineering the integrated domains of NLRs for effector recognition has until now been a primary focus and has included domain swapping, structure-informed mutagenesis, directed evolution, and the incorporation of foreign proteins such as nanobodies (*40*, *42*–*46*). In this study, we demonstrate that this approach can be extended to another class of receptors, the TKPs. The stage is now set for studies to explore engineering of the many recently discovered TKPs in diverse cereal crops to determine their potential for developing new-to-nature disease resistance profiles in agriculture.

## MATERIALS AND METHODS

### Molecular cloning

All plasmids were generated using the Goldengate system using resources from TSL Synbio (*47*) or Infusion cloning (Takara Bio UK LTD, UK). A list of plasmids constructed in this study and domain boundaries used can be found in table S2.

### Production and purification of recombinant proteins

Sanger sequencing–verified constructs were chemically transformed into SHuffle T7 Express C3029 cells (NEB) using the protocol provided by the manufacturer and the transformants were grown on LB agar plates containing 100 μg/mL carbenicillin at 37°C for 16 h. For co-expression, plasmids encoding a 6xHis-tagged effector and untagged HMA domain were simultaneously transformed into SHuffle cells and grown on LB agar plates containing 100 μg/mL carbenicillin and 50 μg/mL kanamycin. Colonies were used to inoculate liquid LB containing the required antibiotics; the resulting cultures were grown overnight at 30°C with shaking. Small-scale overnight cultures were used to inoculate 1 L of liquid LB in a 2-L baffled flask to which the required antibiotics were added, together with 2 mM MgCl_2_ and 200 μL of Antifoam 204 (Sigma-Aldrich Inc., Missouri, USA). These large-scale cultures were incubated at 30°C with shaking at 220 rpm. Cultures were induced to produce the recombinant protein with the addition of isopropyl β-D-1-thiogalactopyranoside (IPTG) to a final concentration of 1 mM once an optical density at 600 nm (OD_600_) of 0.6 was reached, followed by incubation at 18°C with shaking at 220 rpm for 16 h. Cells were harvested by centrifugation at 5000 g for 10 min at 4°C.

Pellets were resuspended in a buffer containing 50 mM HEPES pH 8.0, 300 mM NaCl, 5% (v/v) glycerol, 20 mM imidazole, and 1 mM phenylmethylsulfonyl fluoride (PMSF). The cell pellets were lysed by sonication using an amplitude of 40% (10 seconds on, 20 seconds off). The lysed cells were centrifuged at 30000 g for 30 min at 4°C to clarify the lysate. The protein of interest was purified by immobilized metal affinity chromatography (IMAC) using a 5-mL HisTrap FF nickel column (Cytiva, Massachusetts, USA). The column was washed using a buffer consisting of 50 mM HEPES pH 8.0, 300 mM NaCl, 5% (v/v) glycerol, and 20 mM imidazole prior to elution using an isocratic elution with a buffer composed of 50 mM HEPES pH 8.0, 300 mM NaCl, and 250 mM imidazole. Eluted fractions were analyzed by SDS-PAGE and fractions containing the protein of interest were desalted using a 53-mL SepFast 26/10 1000–6000 Da Desalting Column (BioToolomics Ltd., UK) into 20 mM HEPES pH 7.5, 150 mM NaCl. Fusion proteins were incubated with 3C protease overnight at 4°C; cleavage of the solubility tag was confirmed by SDS-PAGE. The cleaved protein of interest was separated from the N-terminal fusion tag, any uncleaved protein, and 6xHis tagged 3C protease using IMAC and subsequently purified further by size-exclusion chromatography (SEC) using a HiLoad 26/600 Superdex 75 pg column (Cytiva) equilibrated with a buffer consisting of 20 mM HEPES pH 7.5 and 150 mM NaCl. Proteins were concentrated using a 3-kDa or 10-kDa molecular weight cut-off Amicon centrifugal concentrator (Millipore Sigma, Massachusetts, USA), frozen in liquid nitrogen, and stored at −70°C until use.

### Crystallization and crystal optimization

Initial screening to determine the crystallization conditions for co-purified HMA/effector complexes were performed at a concentration of 15.9 mg/mL for Rmo2^HMA^/PBY2, 16.1 mg/mL for Rwt7^HMA^/PWT7, 14.8 mg/mL for Rmo2^HMA+^/PBY2, 20 mg/mL for Rmo2^HMA+^/PBY2/PWT7, 14.6 mg/mL for Rwt7^HMA+^/PBY2, and 16.7 mg/mL for Rwt7^HMA+^/PWT7, in 96-well MRC 2 plates (Hampton Research, California, USA) with incubation at 18°C using the sitting-drop vapor-diffusion method and commercially available sparse matrix screens. Then, 300 nL of each protein solution and 300 nL of reservoir solution were mixed on a sitting-drop well using an Oryx8 robot (Douglas Instruments, UK) and were monitored and imaged using the Rock Imager system (Formulatrix, Massachusetts, USA) over the course of one month. If required, the original crystal hits were optimized in 96-well MRC 2 sitting-drop plates using an Oryx8 robot (Douglas Instruments)

From initial screening, crystals with the best morphology for Rmo2^HMA^/PBY2 were obtained in 2.4 M ammonium sulfate, 0.1 M sodium acetate, pH 4.0 (C3, KISS, JIC in-house screen). Crystal optimization was carried out and the final condition was in 1.6 M ammonium sulfate, 0.1 M sodium acetate, pH 4.2. For both Rwt7^HMA^/PWT7 and Rmo2^HMA+^/PBY2, the best morphology hit was in 0.2 M ammonium sulfate, 0.1 M sodium acetate, pH 4.0, 20% (w/v) polyethylene glycol (PEG) 3350 (E2, KISS, JIC in-house screen). For Rwt7^HMA^/PWT7, the initial hit was optimized, and the final condition was in 0.2 M ammonium sulfate, 0.1 M sodium acetate, pH 4.2, 16% (w/v) PEG 3350. No optimization was required for Rmo2^HMA+^/PBY2. For Rmo2^HMA+^/PBY2/PWT7, crystals were obtained in 0.1 M KBr, 30% (w/v) PEG 2000 MME (G10, JCSG-plus, Molecular Dimensions); the original hit was optimized under the final condition of 0.15 M KBr, 0.1 M MES pH 6.25, 35% (w/v) PEG 2000 MME. The best crystal hit for Rwt7^HMA+^/PBY2 was in 2.4 M ammonium sulfate, 0.1 M MES, pH 6.0 (C11, KISS, JIC in-house screen), while the best hit for Rwt7^HMA+^/PWT7 was in 0.1 M Tris-HCl pH 8.5, 25% (w/v) PEG 6000 (D9, NeXtal PEGs Suite, Molecular Dimensions). No optimization was required for Rwt7^HMA+^/PBY2 or Rwt7^HMA+^/PWT7.

### Data collection and crystal structure determination

Before X-ray data collection, crystals were transferred into a cryoprotectant solution consisting of 20% (v/v) ethylene glycol, vitrified in liquid nitrogen, and shipped to Diamond Light Source for X-ray data collection. Diffraction data were collected at Diamond Light Source on the i04 beamline under the proposal mx32728. The data were scaled and merged by the Aimless program in the CCP4i2 software package (*48*). All structures were solved by molecular replacement using PHASER (*49*). High-confidence AlphaFold2 models of PBY2, PWT7, Rmo2^HMA^, and Rwt7^HMA^ were generated using the ColabFold server (*31*, *32*) and used as search models for molecular replacement. Models were then refined using the REFMAC program in the CCP4i2 package (*50*; model building between refinement rounds was done in COOT (*51*). X-ray data collection and refinement statistics can be found in table S1. Final structures were deposited in the PDB under accession numbers 9TFO, 9TFP, 9TFQ, 9TFR, 9TFS, and 9TFT.

### Analytical size-exclusion chromatography

Purified recombinant proteins (Rmo2^HMA^, Rwt7^HMA^, Rwt7^HMA^-6His, PBY2, PBY2^E42R^, PWT7, PWT7^N39R^) were incubated on ice for at least 1 h alone or in pairs. The incubated proteins were then separated on a Superdex 75 Increase 5/150 GL (Cytiva) column pre-equilibrated in a buffer composed of 20 mM HEPES, pH 7.5, 150 mM NaCl. Samples across the peaks were analyzed by SDS-PAGE and Coomassie staining of the gels.

### Isothermal titration calorimetry (ITC)

For ITC experiments, 300 μL of Rmo2^HMA^, Rwt7^HMA^-6His, Rmo2^HMA+^-6His, or Rwt7^HMA+^-6His at a concentration of 20 μM was loaded into the calorimetric cell. Then, 60 μL of PBY2, PBY2^E42R^, PWT7, or PWT7^N39R^ at 200 μM was loaded into the syringe. Each ITC run was conducted at 25°C in 20 mM HEPES, pH 7.5, 150 mM NaCl, and consisted of a single 0.5-μL syringe injection followed by 18 syringe injections of 2 μL each at 120-second intervals. ITC experiments were performed three times and the data obtained were analyzed using AFFINImeter ITC software (*52*). A global fit across all experimental replicates was performed using a 1:1 binding model. To ensure physical and mathematical consistency across runs, the association constant, binding enthalpy and heat of dilution were treated as shared global parameters linked to a single template experiment. To account for variations in protein concentration, the concentration ratio of the HMA domain in the cell was fixed to 1.

### Protoplast assay

Protoplasts were isolated and transfected according to Knight, Winfield, Goldson, Bargmann and Borrill (*53*). Briefly, leaves from 8–9-day-old wheat seedlings (‘Kronos’ *Triticum durum* cultivar) grown in the dark were harvested and cut into 0.5–1-mm strips in plasmolysis buffer (750 mM mannitol, 1 mM CaCl_2_, 15 mM MES-KOH). Chopped leaves were submerged in enzyme solution (1.5% [w/v] Cellulase RS, 0.5% [w/v] Macerozyme R10, 600 mM mannitol, 10 mM MES-KOH, 1 mM CaCl_2_, 0.1% [w/v] BSA), vacuum-infiltrated, and incubated in the dark for 4 h. Released protoplasts were filtered using a 40-μm filter mesh and centrifuged at 80 g for 3 min with slow acceleration and deceleration settings. The supernatant was replaced with cold W5 buffer (125 mM CaCl_2_, 154 mM NaCl, 2 mM MES-KOH, 5 mM KCl) and the cell suspension was incubated in the dark for 30 min. Protoplasts were centrifuged at 80 g for 3 min with slow acceleration and deceleration settings and the supernatant was replaced with MMG buffer (600 mM mannitol, 15 mM MgCl_2_, 4 mM MES-KOH) to a titer of 3.5 × 10^5^ protoplasts/mL. For transfection, 200 μL of protoplasts were added to tubes containing 10 μg of TKP and effector constructs and 40 μg of the firefly luciferase reporter under the control of the maize *Ubiquitin* promoter. The same volume of PEG solution (40% [w/v] PEG 4000, 80 mM mannitol, 20 mM Ca(NO_3_)_2_) was incubated with the protoplast and DNA mixture for 15 min. The reaction was stopped by adding W5 buffer. Protoplasts were centrifuged at 80 g for 3 min with slow acceleration and deceleration settings and the supernatant was replaced with PIM buffer (600 mM mannitol, 4 mM MES-KOH, 4 mM KCl, 3 mM CaCl_2_); the resulting cell suspension was incubated in the dark at room temperature for 18–20 h. Luciferase activity measurements were conducted following a protocol adapted from Saur, Bauer, Lu and Schulze-Lefert (*34*) using a Luciferase Assay System (Promega E1500). Luciferase activity was measured in a plate reader. Each protoplast experiment comprised six technical replicates, and each experiment was performed three times. Relative luciferase activity, normalized against reactions containing PBY2^E42R^ or PWT7^N39R^, was plotted and statistical analysis was performed using one-way analysis of variance (ANOVA) and *post-hoc* Tukey’s honestly significant difference tests at *P* < 0.05.

### Split GAL4 RUBY assay

Agrobacterium (*Agrobacterium tumefaciens*) cell cultures (GV3101) each carrying an effector construct with the sequence encoding the herpes virus VP16 activation domain in-frame and downstream of the effector gene, or a receptor construct carrying the sequence encoding the yeast GAL4 DNA-binding domain in-frame and downstream of the receptor gene, both under the control of the cauliflower mosaic virus (CaMV) 35S promoter, were resuspended in infiltration buffer (10 mM MES, pH 5.6, 10 mM MgCl_2_, and 200 μM acetosyringone). The cell suspensions were mixed with the final OD_600_ values adjusted to 0.5 for both effector and receptor constructs and 0.1 for an Agrobacterium cell suspension harboring the viral silencing suppressor p19. Leaves were harvested and imaged at 4 days after infiltration. For betalain quantification, leaves were incubated in 100% ethanol until all chlorophyll was removed. Two leaf discs (0.8 cm in diameter) from each infiltration spot were taken and incubated in 500 μL H_2_O for 6 h at room temperature. Then, 200 μL of the extracted betalain solution was placed in wells within a Greiner F-bottom clear plate and absorbance was measured at 538 nm in a microplate reader (SPECTROstar Nano, BMG LABTECH, Germany). All experiments were performed three times.

### Infection assay

Wheat seeds were pre-germinated on wet filter papers. After 24 h, sprouted seeds were sown in the soil in vermiculite supplied with liquid fertilizer in a seedling case (5.5 by 15 by 10 cm) and grown at 22°C in a controlled-environment room under a 12-h light/12-h dark photoperiod provided by fluorescent light bulbs for eight days. Primary leaves from 8-day-old seedlings were fixed onto a hard plastic board with rubber bands just before inoculation. Conidial suspensions (1 × 10^5^ conidia per mL) were prepared as described previously (*54*) and sprayed onto fixed primary leaves using an air compressor. The inoculated leaves were incubated in sealed trays under darkness and humid conditions at 22°C for 24 h, then transferred to dry conditions under a 12-h light/12-h dark photoperiod provided by fluorescent light bulbs and incubated at 22°C for an additional 3 days. Four days after inoculation, symptoms were evaluated based on the size and color of lesions (*55*). The size of lesions was rated on a progressive scale from 0 to 5: 0, no visible infection; 1, pinhead spots; 2, small lesions (<1.5 mm); 3, scattered lesions of intermediate size (<3 mm); 4, large typical lesions; and 5, complete blighting of leaf blades. A disease score comprised a number denoting the lesion size and a letter indicating the lesion color: ‘B’ for brown lesions and ‘G’ for green lesions. Lesion size scores are rated on six progressive grades as follows: 0, no visible infection; 1, pinhead spots; 2, small lesions (<1.5 mm); 3, scattered lesions of intermediate size (<3 mm); 4, large typical lesions; and 5, complete shriveling of leaf blades. Scores 0 to 3 were considered as resistant (R) and 4 to 5 were susceptible (S). Lesion scores were quantified and statistically significant differences (*P* < 0.05) were determined by Wilcoxon rank sum test with Bonferroni correction.

### Immunoblot analysis of protein expression in *Nicotiana benthamiana*

For *N. benthamiana* expression, agrobacterium cultures carrying TKP constructs containing a C-terminal Flag or GAL4-myc tag and effector constructs containing a C-terminal mCherry or VP16 tag were resuspended in infiltration buffer. The cell suspensions were mixed with the final OD_600_ values adjusted to 0.5 for both effector and receptor constructs and 0.1 for an Agrobacterium cell suspension harboring the viral silencing suppressor p19. Leaves were harvested and imaged at 3 days after infiltration. Leaf tissue was frozen in liquid nitrogen and ground into a fine powder. Equal amounts of leaf tissue powder were resuspended in SDS-PAGE sample dye and heat treated. Samples were separated by SDS-PAGE and transferred onto a PVDF membrane for immunoblotting. Effector constructs were probed with anti-RFP antibodies conjugated to horseradish peroxidase (HRP) [Abcam, ab34767, 1:3000 dilution in Tris buffered saline with Tween 20 (TBST)] while receptor constructs were probed with anti-Flag antibodies conjugated to HRP (Cohesion Biosciences, CPA9020, 1:3000 dilution in TBST) or anti-Myc antibodies conjugated to HRP (Thermo Fisher Scientific, A190-105P, 1:3000 dilution in TBST).

## References

[1] N. V. Fedoroff, Food in a future of 10 billion. Agric. Food Secur. 4, 11 (2015).

[2] J. B. Ristaino, P. K. Anderson, D. P. Bebber, K. A. Brauman, N. J. Cunniffe, N. V. Fedoroff, C. Finegold, K. A. Garrett, C. A. Gilligan, C. M. Jones, M. D. Martin, G. K. MacDonald, P. Neenan, A. Records, D. G. Schmale, L. Tateosian, Q. Wei, The persistent threat of emerging plant disease pandemics to global food security. Proc. Natl. Acad. Sci. U.S.A. 118, e2022239118 (2021). 10.1073/pnas.2022239118 PMC8201941 34021073

[3] S. Savary, L. Willocquet, S. J. Pethybridge, P. Esker, N. McRoberts, A. Nelson, The global burden of pathogens and pests on major food crops. Nat. Ecol. Evol. 3, 430–439 (2019). 10.1038/s41559-018-0793-y 30718852

[4] J. D. G. Jones, J. L. Dangl, The plant immune system. Nature 444, 323–329 (2006). 10.1038/nature05286 17108957

[5] M. P. Contreras, D. Lüdke, H. Pai, A. Toghani, S. Kamoun, NLR receptors in plant immunity: Making sense of the alphabet soup. EMBO Rep. 24, e57495 (2023). 10.15252/embr.202357495 PMC10561179 37602936

[6] J. Wang, M. Hu, J. Wang, J. Qi, Z. Han, G. Wang, Y. Qi, H.-W. Wang, J.-M. Zhou, J. Chai, Reconstitution and structure of a plant NLR resistosome conferring immunity. Science 364, eaav5870 (2019). 10.1126/science.aav5870 30948527

[7] A. Förderer, E. Li, A. W. Lawson, Y.-n. Deng, Y. Sun, E. Logemann, X. Zhang, J. Wen, Z. Han, J. Chang, Y. Chen, P. Schulze-Lefert, J. Chai, A wheat resistosome defines common principles of immune receptor channels. Nature 610, 532–539 (2022). 10.1038/s41586-022-05231-w PMC9581773 36163289

[8] R. Martin, T. Qi, H. Zhang, F. Liu, M. King, C. Toth, E. Nogales, B. J. Staskawicz, Structure of the activated ROQ1 resistosome directly recognizing the pathogen effector XopQ. Science 370, eabd9993 (2020). 10.1126/science.abd9993 PMC7995448 33273074

[9] S. Ma, D. Lapin, L. Liu, Y. Sun, W. Song, X. Zhang, E. Logemann, D. Yu, J. Wang, J. Jirschitzka, Z. Han, P. Schulze-Lefert, J. E. Parker, J. Chai, Direct pathogen-induced assembly of an NLR immune receptor complex to form a holoenzyme. Science 370, eabe3069 (2020). 10.1126/science.abe3069 33273071

[10] J. Madhuprakash, A. Toghani, M. P. Contreras, A. Posbeyikian, J. Richardson, J. Kourelis, T. O. Bozkurt, M. W. Webster, S. Kamoun, A disease resistance protein triggers oligomerization of its NLR helper into a hexameric resistosome to mediate innate immunity. Sci. Adv. 10, eadr2594 (2024). 10.1126/sciadv.adr2594 PMC11540030 39504373

[11] P. M. Dracatos, J. Lu, J. Sánchez-Martín, B. B. H. Wulff, Resistance that stacks up: Engineering rust and mildew disease control in the cereal crops wheat and barley. Plant Biotechnol. J. 21, 1938–1951 (2023). 10.1111/pbi.14106 PMC10502761 37494504

[12] R. Brueggeman, N. Rostoks, D. Kudrna, A. Kilian, F. Han, J. Chen, A. Druka, B. Steffenson, A. Kleinhofs, The barley stem rust-resistance gene *Rpg1* is a novel disease-resistance gene with homology to receptor kinases. Proc. Natl. Acad. Sci. U.S.A. 99, 9328–9333 (2002). 10.1073/pnas.142284999 PMC123140 12077318

[13] V. Klymiuk, E. Yaniv, L. Huang, D. Raats, A. Fatiukha, S. Chen, L. Feng, Z. Frenkel, T. Krugman, G. Lidzbarsky, W. Chang, M. J. Jääskeläinen, C. Schudoma, L. Paulin, P. Laine, H. Bariana, H. Sela, K. Saleem, C. K. Sørensen, M. S. Hovmøller, A. Distelfeld, B. Chalhoub, J. Dubcovsky, A. B. Korol, A. H. Schulman, T. Fahima, Cloning of the wheat Yr15 resistance gene sheds light on the plant tandem kinase-pseudokinase family. Nat. Commun. 9, 3735 (2018). 10.1038/s41467-018-06138-9 PMC6170490 30282993

[14] P. Lu, L. Guo, Z. Wang, B. Li, J. Li, Y. Li, D. Qiu, W. Shi, L. Yang, N. Wang, G. Guo, J. Xie, Q. Wu, Y. Chen, M. Li, H. Zhang, L. Dong, P. Zhang, K. Zhu, D. Yu, Y. Zhang, K. R. Deal, N. Huo, C. Liu, M.-C. Luo, J. Dvorak, Y. Q. Gu, H. Li, Z. Liu, A rare gain of function mutation in a wheat tandem kinase confers resistance to powdery mildew. Nat. Commun. 11, 680 (2020). 10.1038/s41467-020-14294-0 PMC6997164 32015344

[15] S. Chen, M. N. Rouse, W. Zhang, X. Zhang, Y. Guo, J. Briggs, J. Dubcovsky, Wheat gene Sr60 encodes a protein with two putative kinase domains that confers resistance to stem rust. New Phytol. 225, 948–959 (2020). 10.1111/nph.16169 31487050

[16] J. Sánchez-Martín, V. Widrig, G. Herren, T. Wicker, H. Zbinden, J. Gronnier, L. Spörri, C. R. Praz, M. Heuberger, M. C. Kolodziej, J. Isaksson, B. Steuernagel, M. Karafiátová, J. Doležel, C. Zipfel, B. Keller, Wheat Pm4 resistance to powdery mildew is controlled by alternative splice variants encoding chimeric proteins. Nat. Plants 7, 327–341 (2021). 10.1038/s41477-021-00869-2 PMC7610370 33707738

[17] G. Yu, O. Matny, N. Champouret, B. Steuernagel, M. J. Moscou, I. Hernández-Pinzón, P. Green, S. Hayta, M. Smedley, W. Harwood, N. Kangara, Y. Yue, C. Gardener, M. J. Banfield, P. D. Olivera, C. Welchin, J. Simmons, E. Millet, A. Minz-Dub, M. Ronen, R. Avni, A. Sharon, M. Patpour, A. F. Justesen, M. Jayakodi, A. Himmelbach, N. Stein, S. Wu, J. Poland, J. Ens, C. Pozniak, M. Karafiátová, I. Molnár, J. Doležel, E. R. Ward, T. L. Reuber, J. D. G. Jones, M. Mascher, B. J. Steffenson, B. B. H. Wulff, Aegilops sharonensis genome-assisted identification of stem rust resistance gene Sr62. Nat. Commun. 13, 1607 (2022). 10.1038/s41467-022-29132-8 PMC8956640 35338132

[18] K. Gaurav, S. Arora, P. Silva, J. Sánchez-Martín, R. Horsnell, L. Gao, G. S. Brar, V. Widrig, W. John Raupp, N. Singh, S. Wu, S. M. Kale, C. Chinoy, P. Nicholson, J. Quiroz-Chávez, J. Simmonds, S. Hayta, M. A. Smedley, W. Harwood, S. Pearce, D. Gilbert, N. Kangara, C. Gardener, M. Forner-Martínez, J. Liu, G. Yu, S. A. Boden, A. Pascucci, S. Ghosh, A. N. Hafeez, T. O’Hara, J. Waites, J. Cheema, B. Steuernagel, M. Patpour, A. F. Justesen, S. Liu, J. C. Rudd, R. Avni, A. Sharon, B. Steiner, R. P. Kirana, H. Buerstmayr, A. A. Mehrabi, F. Y. Nasyrova, N. Chayut, O. Matny, B. J. Steffenson, N. Sandhu, P. Chhuneja, E. Lagudah, A. F. Elkot, S. Tyrrell, X. Bian, R. P. Davey, M. Simonsen, L. Schauser, V. K. Tiwari, H. Randy Kutcher, P. Hucl, A. Li, D.-C. Liu, L. Mao, S. Xu, G. Brown-Guedira, J. Faris, J. Dvorak, M.-C. Luo, K. Krasileva, T. Lux, S. Artmeier, K. F. X. Mayer, C. Uauy, M. Mascher, A. R. Bentley, B. Keller, J. Poland, B. B. H. Wulff, Population genomic analysis of *Aegilops tauschii* identifies targets for bread wheat improvement. Nat. Biotechnol. 40, 422–431 (2022). 10.1038/s41587-021-01058-4 PMC8926922 34725503

[19] Y. Wang, M. Abrouk, S. Gourdoupis, D.-H. Koo, M. Karafiátová, I. Molnár, K. Holušová, J. Doležel, N. Athiyannan, E. Cavalet-Giorsa, Ł. Jaremko, J. Poland, S. G. Krattinger, An unusual tandem kinase fusion protein confers leaf rust resistance in wheat. Nat. Genet. 55, 914–920 (2023). 10.1038/s41588-023-01401-2 PMC10260399 37217716

[20] G. Yu, O. Matny, S. Gourdoupis, N. Rayapuram, F. R. Aljedaani, Y. L. Wang, T. Nürnberger, R. Johnson, E. E. Crean, I. M. L. Saur, C. Gardener, Y. Yue, N. Kangara, B. Steuernagel, S. Hayta, M. Smedley, W. Harwood, M. Patpour, S. Wu, J. Poland, J. D. G. Jones, T. L. Reuber, M. Ronen, A. Sharon, M. N. Rouse, S. Xu, K. Holušová, J. Bartoš, I. Molnár, M. Karafiátová, H. Hirt, I. Blilou, Ł. Jaremko, J. Doležel, B. J. Steffenson, B. B. H. Wulff, The wheat stem rust resistance gene Sr43 encodes an unusual protein kinase. Nat. Genet. 55, 921–926 (2023). 10.1038/s41588-023-01402-1 PMC10260397 37217714

[21] S. Arora, A. Steed, R. Goddard, K. Gaurav, T. O’Hara, A. Schoen, N. Rawat, A. F. Elkot, A. V. Korolev, C. Chinoy, M. H. Nicholson, S. Asuke, R. Antoniou-Kourounioti, B. Steuernagel, G. Yu, R. Awal, M. Forner-Martínez, L. Wingen, E. Baggs, J. Clarke, D. G. O. Saunders, K. V. Krasileva, Y. Tosa, J. D. G. Jones, V. K. Tiwari, B. B. H. Wulff, P. Nicholson, A wheat kinase and immune receptor form host-specificity barriers against the blast fungus. Nat. Plants 9, 385–392 (2023). 10.1038/s41477-023-01357-5 PMC10027608 36797350

[22] H. Li, W. Men, C. Ma, Q. Liu, Z. Dong, X. Tian, C. Wang, C. Liu, H. S. Gill, P. Ma, Z. Zhang, B. Liu, Y. Zhao, S. K. Sehgal, W. Liu, Wheat powdery mildew resistance gene Pm13 encodes a mixed lineage kinase domain-like protein. Nat. Commun. 15, 2449 (2024). 10.1038/s41467-024-46814-7 PMC10951266 38503771

[23] Y. Zhao, Z. Dong, J. Miao, Q. Liu, C. Ma, X. Tian, J. He, H. Bi, W. Yao, T. Li, H. S. Gill, Z. Zhang, A. Cao, B. Liu, H. Li, S. K. Sehgal, W. Liu, Pm57 from *Aegilops searsii* encodes a tandem kinase protein and confers wheat powdery mildew resistance. Nat. Commun. 15, 4796 (2024). 10.1038/s41467-024-49257-2 PMC11153570 38839783

[24] S. Asuke, A. G. Tagle, G.-S. Hyon, S. Koizumi, T. Murakami, A. Horie, D. Niwamoto, E. Katayama, M. Shibata, Y. Takahashi, M. T. Islam, Y. Matsuoka, N. Yamaji, M. Shimizu, R. Terauchi, H. Hisano, K. Sato, Y. Tosa, Evolution of HMA-integrated tandem kinases accompanied by expansion of target pathogens. biorxiv 2025.12.15.692859 [Preprint] (2025). 10.64898/2025.12.15.692859.

[25] R. Chen, J. Chen, O. R. Powell, M. A. Outram, T. Arndell, K. Gajendiran, Y. L. Wang, J. Lubega, Y. Xu, M. A. Ayliffe, C. Blundell, M. Figueroa, J. Sperschneider, T. Vanhercke, K. Kanyuka, D. Tang, G. Zhong, C. Gardener, G. Yu, S. Gourdoupis, Ł. Jaremko, O. Matny, B. J. Steffenson, W. H. P. Boshoff, W. B. Meyer, S. T. Arold, P. N. Dodds, B. B. H. Wulff, A wheat tandem kinase activates an NLR to trigger immunity. Science 387, 1402–1408 (2025). 10.1126/science.adp5034 40146821

[26] P. Lu, G. Zhang, J. Li, Z. Gong, G. Wang, L. Dong, H. Zhang, G. Guo, M. Su, K. Wang, Y. Wang, K. Zhu, Q. Wu, Y. Chen, M. Li, B. Huang, B. Li, W. Li, L. Dong, Y. Hou, X. Cui, H. Fu, D. Qiu, C. Yuan, H. Li, J.-M. Zhou, G.-Z. Han, Y. Chen, Z. Liu, A wheat tandem kinase and NLR pair confers resistance to multiple fungal pathogens. Science 387, 1418–1424 (2025). 10.1126/science.adp5469 40146830

[27] Y.-C. Sung, Y. Li, Z. Bernasconi, S. Baik, S. Asuke, B. Keller, T. Fahima, G. Coaker, Wheat tandem kinase RWT4 directly binds a fungal effector to activate defense. Nat. Genet. 57, 1238–1249 (2025). 10.1038/s41588-025-02162-w PMC12282664 40229601

[28] H. Kanzaki, K. Yoshida, H. Saitoh, K. Fujisaki, A. Hirabuchi, L. Alaux, E. Fournier, D. Tharreau, R. Terauchi, Arms race co-evolution of *Magnaporthe oryzae* AVR-Pik and rice Pik genes driven by their physical interactions. Plant J. 72, 894–907 (2012). 10.1111/j.1365-313X.2012.05110.x 22805093

[29] A. Maqbool, H. Saitoh, M. Franceschetti, C. E. M. Stevenson, A. Uemura, H. Kanzaki, S. Kamoun, R. Terauchi, M. J. Banfield, Structural basis of pathogen recognition by an integrated HMA domain in a plant NLR immune receptor. eLife 4, e08709 (2015). 10.7554/eLife.08709 PMC4547098 26304198

[30] T. Reveguk, A. Fatiukha, E. Potapenko, I. Reveguk, H. Sela, V. Klymiuk, Y. Li, C. Pozniak, T. Wicker, G. Coaker, T. Fahima, Tandem kinase proteins across the plant kingdom. Nat. Genet. 57, 254–262 (2025). 10.1038/s41588-024-02032-x PMC12282663 39779952

[31] J. Jumper, R. Evans, A. Pritzel, T. Green, M. Figurnov, O. Ronneberger, K. Tunyasuvunakool, R. Bates, A. Žídek, A. Potapenko, A. Bridgland, C. Meyer, S. A. A. Kohl, A. J. Ballard, A. Cowie, B. Romera-Paredes, S. Nikolov, R. Jain, J. Adler, T. Back, S. Petersen, D. Reiman, E. Clancy, M. Zielinski, M. Steinegger, M. Pacholska, T. Berghammer, S. Bodenstein, D. Silver, O. Vinyals, A. W. Senior, K. Kavukcuoglu, P. Kohli, D. Hassabis, Highly accurate protein structure prediction with AlphaFold. Nature 596, 583–589 (2021). 10.1038/s41586-021-03819-2 PMC8371605 34265844

[32] M. Mirdita, K. Schütze, Y. Moriwaki, L. Heo, S. Ovchinnikov, M. Steinegger, ColabFold: Making protein folding accessible to all. Nat. Methods *19*, 679–682 (2022). 10.1038/s41592-022-01488-1 PMC9184281 35637307

[33] J. Chen, M. Luo, P. Hands, V. Rolland, J. Zhang, Z. Li, M. Outram, P. Dodds, M. Ayliffe, A split GAL4 RUBY assay for visual in planta detection of protein–protein interactions. Plant J. 114, 1209–1226 (2023). 10.1111/tpj.16234 37323061

[34] I. M. L. Saur, S. Bauer, X. Lu, P. Schulze-Lefert, A cell death assay in barley and wheat protoplasts for identification and validation of matching pathogen AVR effector and plant NLR immune receptors. Plant Methods 15, 118 (2019). 10.1186/s13007-019-0502-0 PMC6813131 31666804

[35] A. Mirlohi, R. Brueggeman, T. Drader, J. Nirmala, B. J. Steffenson, A. Kleinhofs, Allele sequencing of the barley stem rust resistance gene Rpg1 identifies regions relevant to disease resistance. Phytopathology 98, 910–918 (2008). 10.1094/PHYTO-98-8-0910 18943209

[36] N. T. T. Nga, Y. Inoue, I. Chuma, G.-S. Hyon, K. Okada, T. T. P. Vy, M. Kusaba, Y. Tosa, Identification of a novel locus Rmo2 conditioning resistance in barley to host-specific subgroups of *Magnaporthe oryzae*. Phytopathology 102, 674–682 (2012). 10.1094/PHYTO-09-11-0256 22667446

[37] S. Cesari, Y. Xi, N. Declerck, V. Chalvon, L. Mammri, M. Pugnière, C. Henriquet, K. de Guillen, V. Chochois, A. Padilla, T. Kroj, New recognition specificity in a plant immune receptor by molecular engineering of its integrated domain. Nat. Commun. 13, 1524 (2022). 10.1038/s41467-022-29196-6 PMC8938504 35314704

[38] J. Nirmala, R. Brueggeman, C. Maier, C. Clay, N. Rostoks, C. G. Kannangara, D. von Wettstein, B. J. Steffenson, A. Kleinhofs, Subcellular localization and functions of the barley stem rust resistance receptor-like serine/threonine-specific protein kinase Rpg1. Proc. Natl. Acad. Sci. U.S.A. 103, 7518–7523 (2006). 10.1073/pnas.0602379103 PMC1464370 16648249

[39] I. Saado, H. J. Brabham, J. W. Bennett, A. H. C. Lam, I. Hernández-Pinzón, M. J. Moscou, J. C. De la Concepcion, M. J. Banfield, Engineering an Exo70 integrated domain of a barley NLR for improved blast resistance. bioRxiv 2024.12.24.630226 [Preprint] (2024). 10.1101/2024.12.24.630226. PMC13291817 42263189

[40] R. Zdrzałek, Y. Xi, T. Langner, A. R. Bentham, Y. Petit-Houdenot, J. C. De la Concepcion, A. Harant, M. Shimizu, V. Were, N. J. Talbot, R. Terauchi, S. Kamoun, M. J. Banfield, Bioengineering a plant NLR immune receptor with a robust binding interface toward a conserved fungal pathogen effector. Proc. Natl. Acad. Sci. U.S.A. 121, e2402872121 (2024). 10.1073/pnas.2402872121 PMC11252911 38968126

[41] K. Seong, W. Wei, S. C. Sent, B. Vega, A. Dee, G. Ramirez-Bernardino, R. Kumar, L. Parra, I. M. Saur, K. Krasileva, Resurrection of the plant immune receptor Sr50 to overcome pathogen immune evasion. bioRxiv 2024.08.07.607039 [Preprint] (2025). 10.1101/2024.08.07.607039.

[42] J. Kourelis, C. Marchal, A. Posbeyikian, A. Harant, S. Kamoun, NLR immune receptor–nanobody fusions confer plant disease resistance. Science 379, 934–939 (2023). 10.1126/science.abn4116 36862785

[43] J. H. R. Maidment, M. Franceschetti, A. Maqbool, H. Saitoh, C. Jantasuriyarat, S. Kamoun, R. Terauchi, M. J. Banfield, Multiple variants of the fungal effector AVR-Pik bind the HMA domain of the rice protein OsHIPP19, providing a foundation to engineer plant defense. J. Biol. Chem. 296, 100371 (2021). 10.1016/j.jbc.2021.100371 PMC7961100 33548226

[44] J. H. R. Maidment, M. Shimizu, A. R. Bentham, S. Vera, M. Franceschetti, A. Longya, C. E. M. Stevenson, J. C. De la Concepcion, A. Białas, S. Kamoun, R. Terauchi, M. J. Banfield, Effector target-guided engineering of an integrated domain expands the disease resistance profile of a rice NLR immune receptor. eLife 12, e81123 (2023). 10.7554/eLife.81123 PMC10195085 37199729

[45] E. Y. Rim, O. D. Garrett, A. J. Howard, Y. Shim, Y. Li, J. E. Van Dyke, R. C. Packer, N. Ho, R. Jain, V. Stewart, S. P. Dinesh-Kumar, J. H. Notwell, P. C. Ronald, Directed evolution of a plant immune receptor for broad spectrum effector recognition. bioRxiv 2024.09.30.614878 [Preprint] (2025). 10.1101/2024.09.30.614878.

[46] R. Zdrzałek, C. Stone, J. C. De la Concepcion, M. J. Banfield, A. R. Bentham, Pathways to engineering plant intracellular NLR immune receptors. Curr. Opin. Plant Biol. 74, 102380 (2023). 10.1016/j.pbi.2023.102380 37187111

[47] A. R. Bentham, M. Youles, M. N. Mendel, F. A. Varden, J. C. D. l. Concepcion, M. J. Banfield, pOPIN-GG: A resource for modular assembly in protein expression vectors. bioRxiv 2021.08.10.455798 [Preprint] (2021). 10.1101/2021.08.10.455798.

[48] L. Potterton, J. Agirre, C. Ballard, K. Cowtan, E. Dodson, P. R. Evans, H. T. Jenkins, R. Keegan, E. Krissinel, K. Stevenson, A. Lebedev, S. J. McNicholas, R. A. Nicholls, M. Noble, N. S. Pannu, C. Roth, G. Sheldrick, P. Skubak, J. Turkenburg, V. Uski, F. von Delft, D. Waterman, K. Wilson, M. Winn, M. Wojdyr, CCP4i2: The new graphical user interface to the CCP4 program suite. Acta Crystallogr. D Struct. Biol. 74, 68–84 (2018). 10.1107/S2059798317016035 PMC5947771 29533233

[49] A. J. McCoy, R. W. Grosse-Kunstleve, P. D. Adams, M. D. Winn, L. C. Storoni, R. J. Read, Phaser crystallographic software. J. Appl. Cryst. 40, 658–674 (2007). 10.1107/S0021889807021206 PMC2483472 19461840

[50] G. N. Murshudov, P. Skubák, A. A. Lebedev, N. S. Pannu, R. A. Steiner, R. A. Nicholls, M. D. Winn, F. Long, A. A. Vagin, REFMAC5 for the refinement of macromolecular crystal structures. Acta Crystallogr. D Biol. Crystallogr. 67, 355–367 (2011). 10.1107/S0907444911001314 PMC3069751 21460454

[51] P. Emsley, K. Cowtan, Coot: Model-building tools for molecular graphics. Acta Crystallogr. D Biol. Crystallogr. 60, 2126–2132 (2004). 10.1107/S0907444904019158 15572765

[52] Á. Piñeiro, E. Muñoz, J. Sabín, M. Costas, M. Bastos, A. Velázquez-Campoy, P. F. Garrido, P. Dumas, E. Ennifar, L. García-Río, J. Rial, D. Pérez, P. Fraga, A. Rodríguez, C. Cotelo, AFFINImeter: A software to analyze molecular recognition processes from experimental data. Anal. Biochem. 577, 117–134 (2019). 10.1016/j.ab.2019.02.031 30849378

[53] E. Knight, M. Winfield, A. Goldson, B. Bargmann, P. Borrill, B. Bargmann, Identifying direct TARGETs of transcription factors in wheat. *protocols.io* (2025); 10.17504/protocols.io.q26g75bj1lwz/v2.

[54] A. G. Tagle, I. Chuma, Y. Tosa, Rmg7, a New Gene for Resistance to Triticum Isolates of Pyricularia oryzae Identified in Tetraploid Wheat. Phytopathology 105, 495–499 (2015). 10.1094/PHYTO-06-14-0182-R 25870924

[55] G.-S. Hyon, N. T. T. Nga, I. Chuma, Y. Inoue, H. Asano, N. Murata, M. Kusaba, Y. Tosa, Characterization of interactions between barley and various host-specific subgroups of *Magnaporthe oryzae* and *M. grisea*. J. Gen. Plant Pathol. 78, 237–246 (2012).

