# Supplementary material for: Engineering plant tandem kinase immune receptors expands effector recognition profiles
Source: Sci Adv. 2026 Sep 23;12(39):eaef2426. doi: 10.1126/sciadv.aef2426 (PMC13600239; doi:10.1126/sciadv.aef2426)
Supplement: Supplementary file 1 [file sciadv.aef2426_sm.pdf]

Science Advances
AAAS

Supplementary Materials for

Engineering plant tandem kinase immune receptors expands effector recognition profiles

Daniel S. Yu *et al.*

Corresponding author: Mark J. Banfield, mark.banfield@jic.ac.uk

Sci. Adv. **12**, eaef2426 (2026)
DOI: 10.1126/sciadv.aef2426

This PDF file includes:

Figs. S1 to S14
Tables S1 and S2

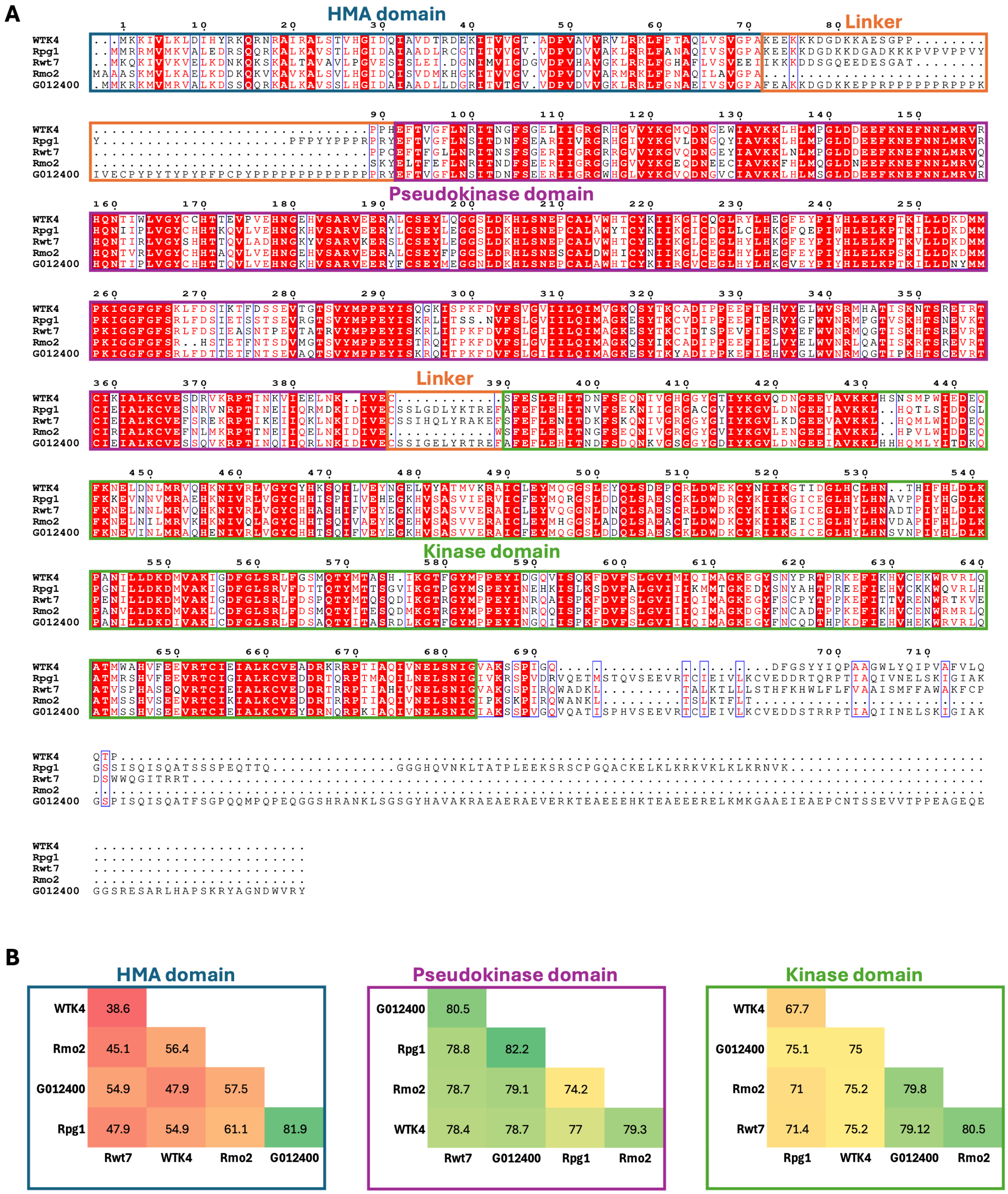

Fig. S1. Sequence analysis of HMA-TKPs. (A) Amino acid sequence alignment of Rmo2, Rwt7, Rpg1, WTK4 and G012400. The integrated HMA, pseudokinase, and kinase domains are highlighted in blue, purple and green, respectively. Linker regions between the domains are highlighted in orange. Domain boundaries were defined structurally using structural models generated in AF3. The alignment was generated using the ESPript 3.0 server. **(B)** Sequence identity matrices of the HMA domain, pseudokinase and kinase domains of HMA-TKPs. Percent identity was calculated in the MUSCLE alignment server.

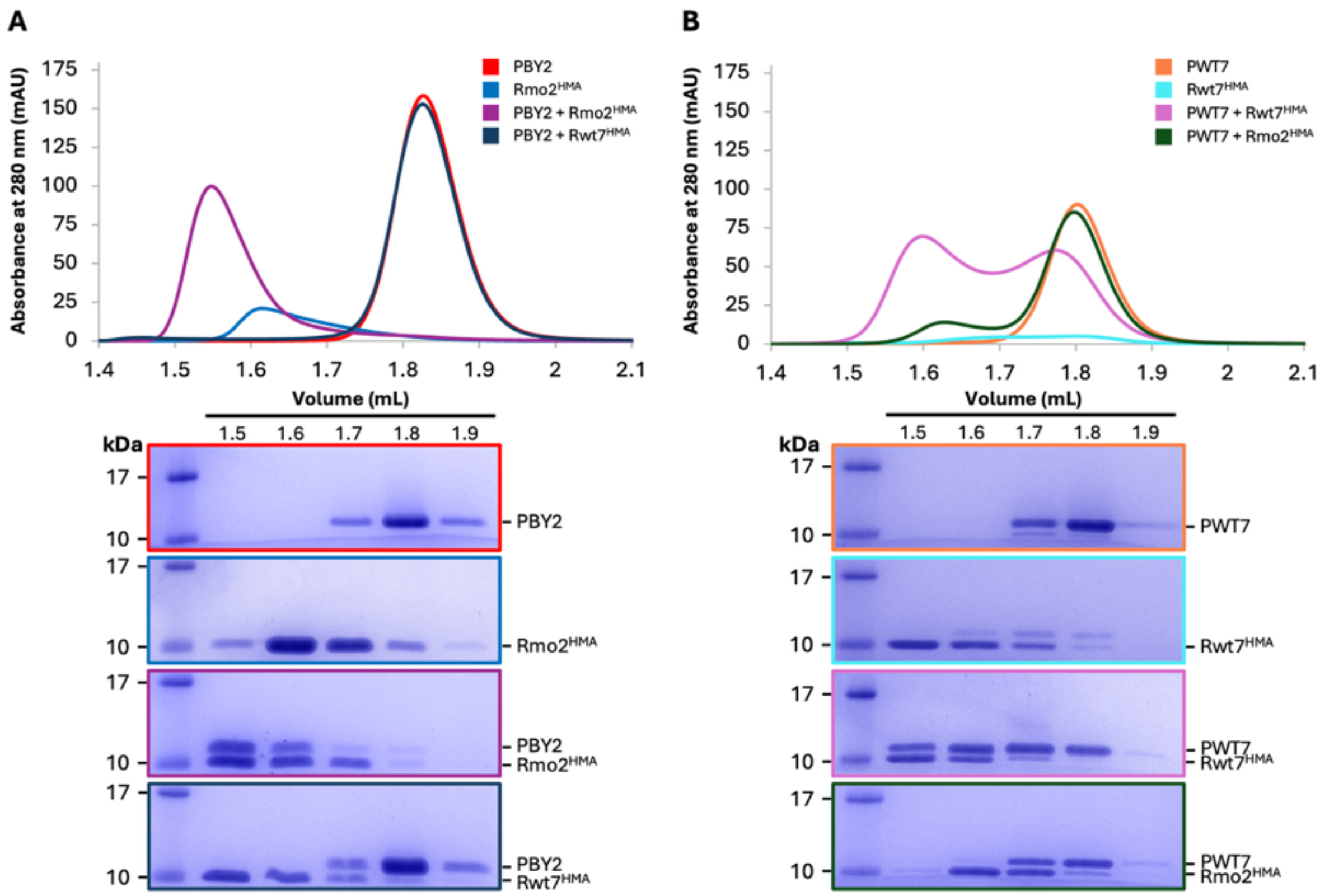

Fig. S2. Analytical size-exclusion chromatography of integrated HMA domains with their cognate and non-cognate effectors. (A) Representative size-exclusion chromatograms of PBY2 alone (red), Rmo2^{HMA} alone (blue), PBY2 and Rmo2^{HMA} (purple), PBY2 and Rwt7^{HMA} (navy), and **(B)** PWT7 alone (orange), Rwt7^{HMA} alone (cyan), PWT7 and Rwt7^{HMA} (pink), and PWT7 and Rmo2^{HMA} (dark green). Proteins were incubated on ice for one hour and then separated on a Superdex 75 Increase 5/150 GL column. Coomassie-stained SDS-PAGE gels (bottom panels) depicting samples taken from 100-µL fractions corresponding to the volumes indicated above the gels.

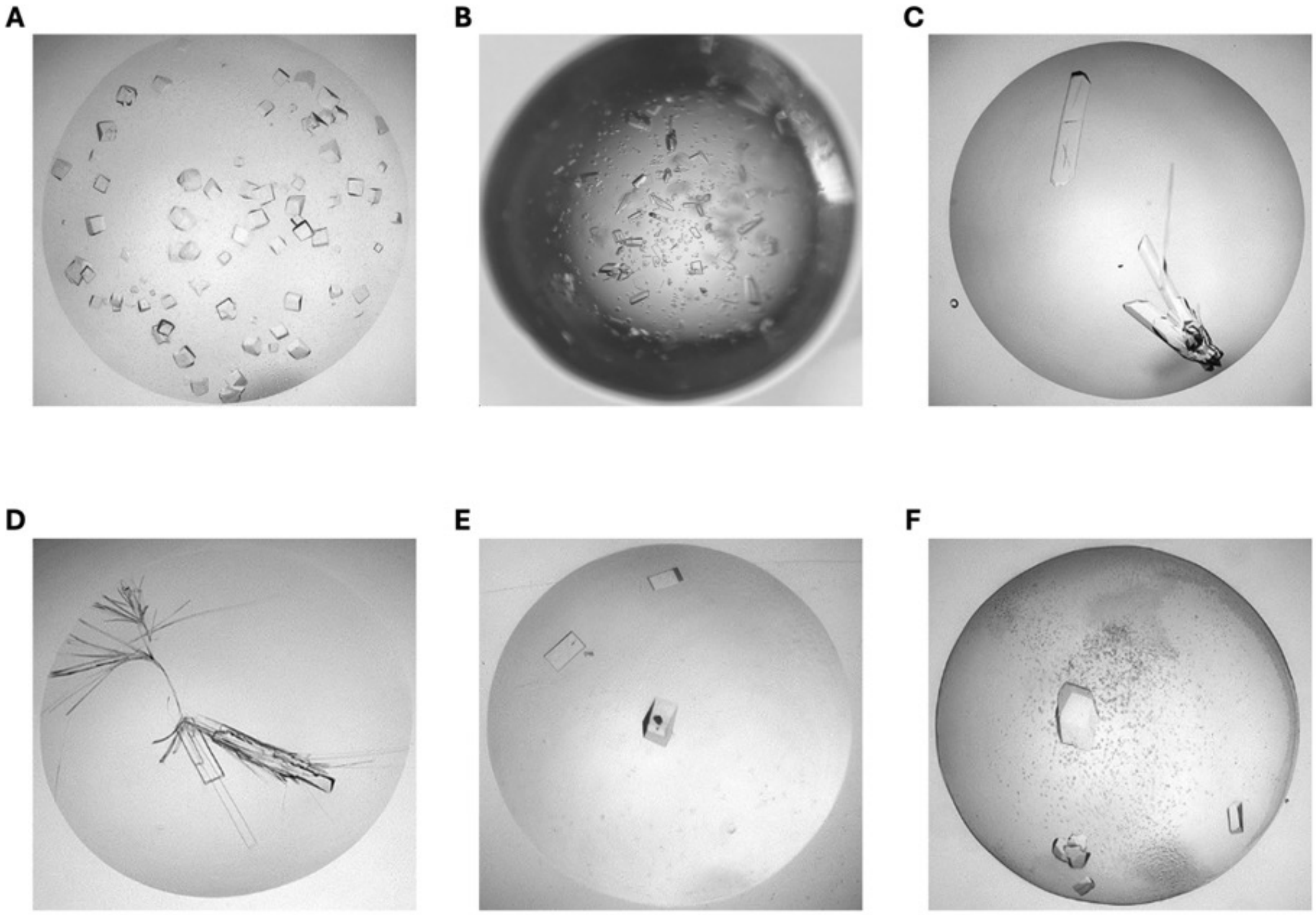

Fig. S3. Crystallization of HMA/effector complexes. Optimized protein crystals of **(A)** $Rmo2^{HMA}$/PBY2, **(B)** $Rwt7^{HMA}$/PWT7, **(C)** $Rmo2^{HMA+}$/PBY2, **(D)** $Rmo2^{HMA+}$/PBY2/PWT7, **(E)** $Rwt7^{HMA+}$/PBY2 and **(F)** $Rwt7^{HMA+}$/PWT7.

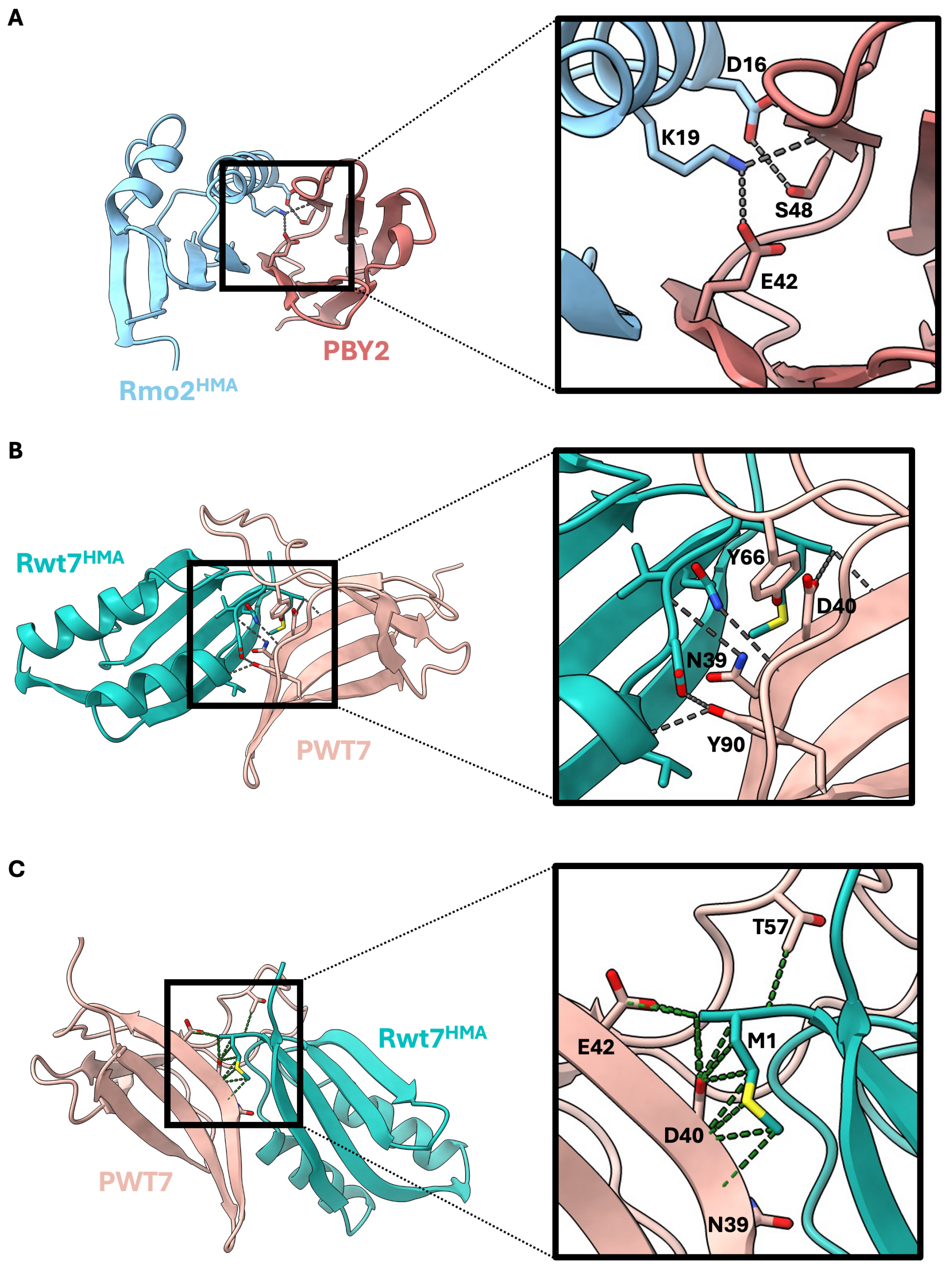

Fig. S4. Identification of residues within effectors that contribute to their interaction with the integrated HMA domain of Rmo2 or Rwt7. (A) Hydrogen bonds between Rmo2^{HMA}/PBY2 and **(B)** Rwt7^{HMA}/PWT7. Interacting residues are shown as sticks. Key residues within the effector proteins are labeled. **(C)** Extensive contacts of the M1 residue from Rwt7^{HMA} with PWT7 residues.

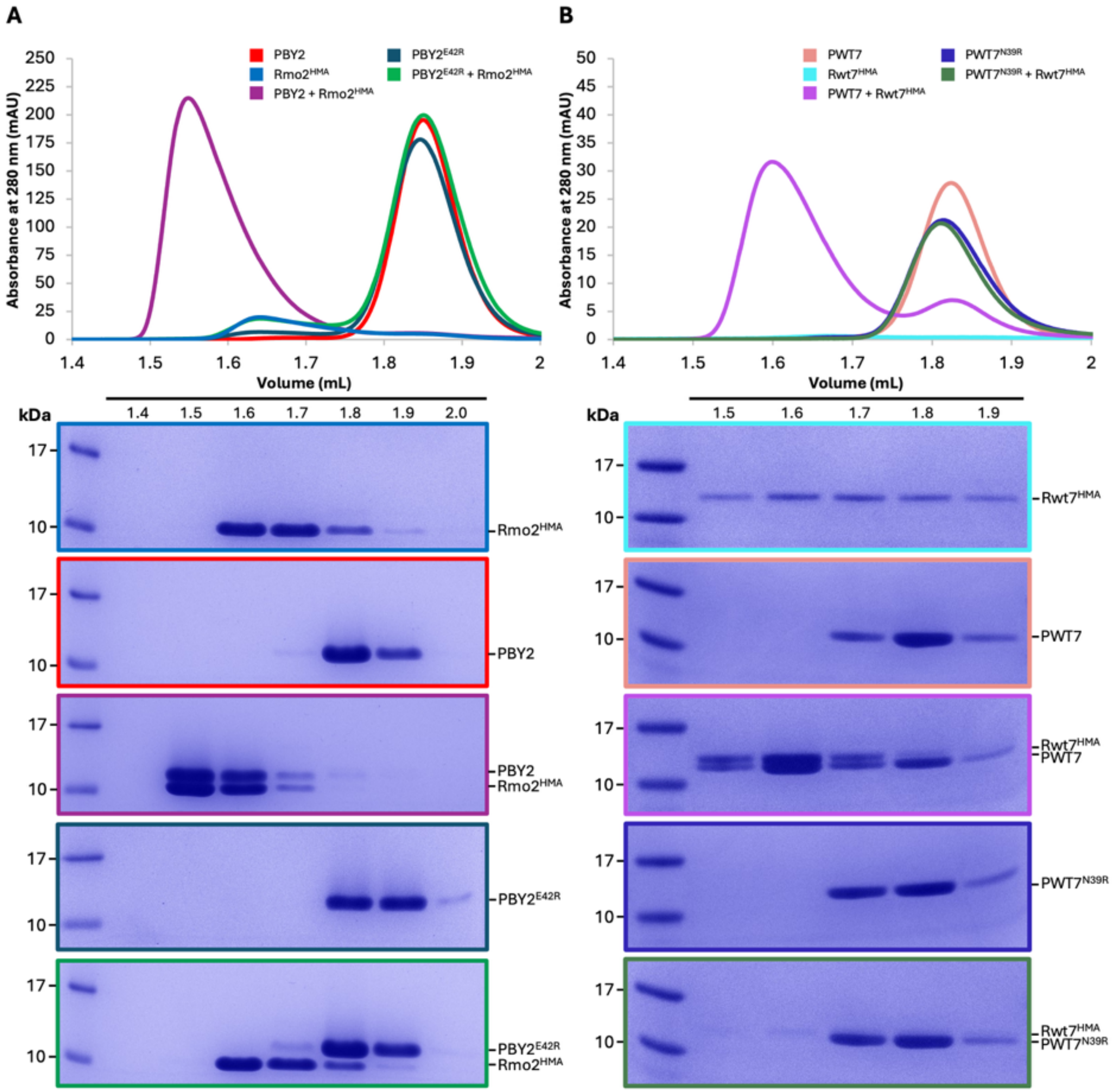

Fig. S5. Structure-informed effector mutants do not interact with integrated HMA domains by analytical size-exclusion chromatography. (A) Size-exclusion chromatograms of Rmo2^{HMA} alone (blue), PBY2 alone (red), Rmo2^{HMA} and PBY2 (purple), PBY2^{E42R} alone (navy), Rmo2^{HMA} and PBY2^{E42R} (green), and **(B)** Rwt7^{HMA} alone (cyan), PWT7 alone (orange), Rwt7^{HMA} and PWT7 (pink), PWT7^{N39R} alone (dark blue) and Rwt7^{HMA} and PWT7^{N39R} (dark green). Proteins were incubated on ice for one hour and then separated on a Superdex 75 Increase 5/150 GL column. Coomassie-stained SDS-PAGE gels (bottom panels) depicting samples taken from 100-µL fractions corresponding to the volumes indicated above the gels.

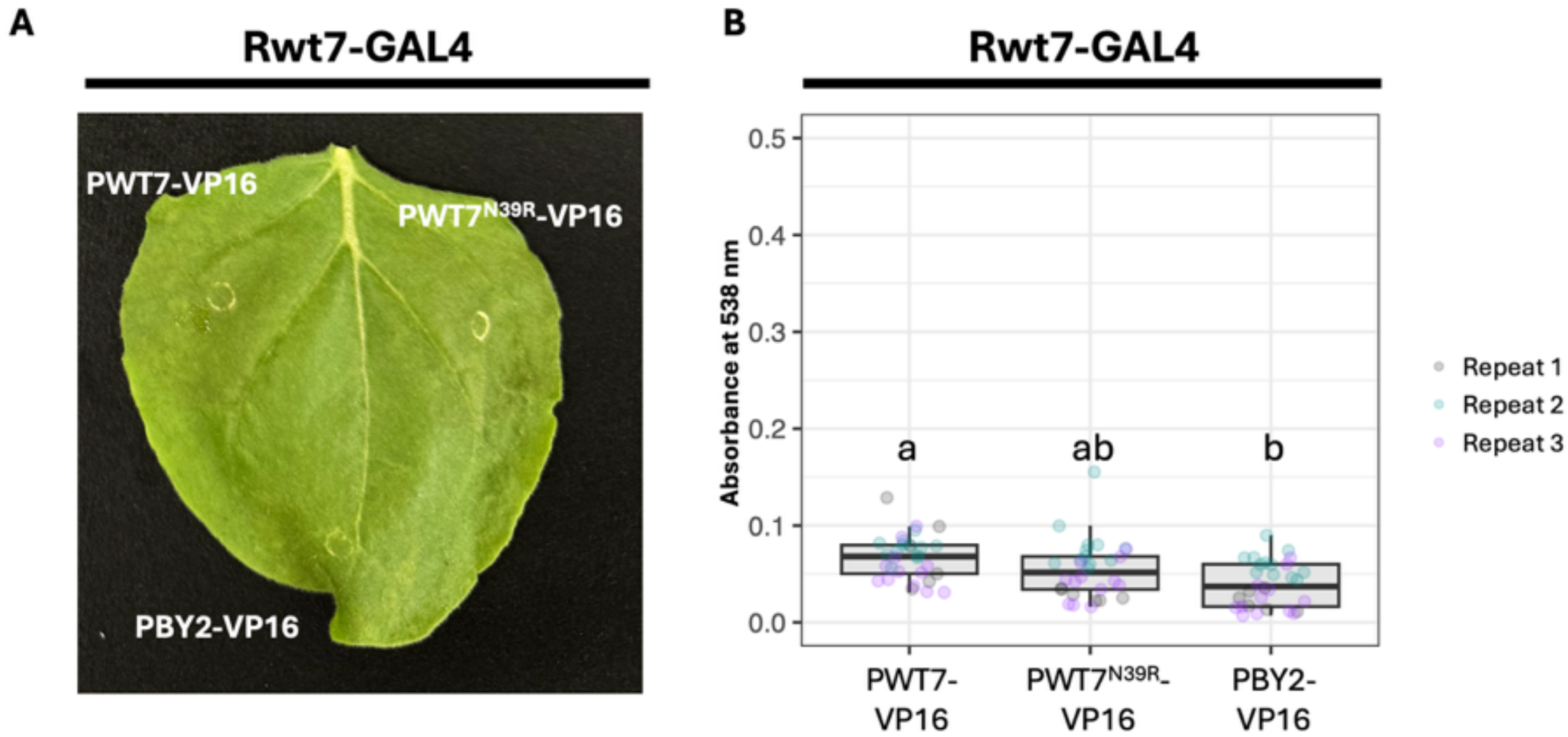

Fig. S6. Limited betalain is produced with Rwt7-GAL4 using the split GAL4 RUBY assay. (A) Representative *Nicotiana benthamiana* leaves, showing little betalain production following transient co-expression of *Rwt7-GAL4* (encoding a fusion of Rwt7 with the DNA-binding domain of yeast GAL4 at its C terminus) with *PWT7-VP16*, *PWT7^{N39R}-VP16*, or *PBY2-VP16* (encoding each effector fused to VP16 at their C termini). All expression cassettes were under the control of the cauliflower mosaic virus (CaMV) 35S promoter. Leaves were harvested 4 days after co-infiltration and chlorophyll was cleared with ethanol. Betalain was extracted from each infiltrated spot and absorbance was measured at 538 nm. Each experiment consisted of 6 or 12 replicates, performed three times independently. Different letters indicate significant differences, as determined by one-way ANOVA and *post-hoc* Tukey's honestly significant difference tests at $P < 0.05$.

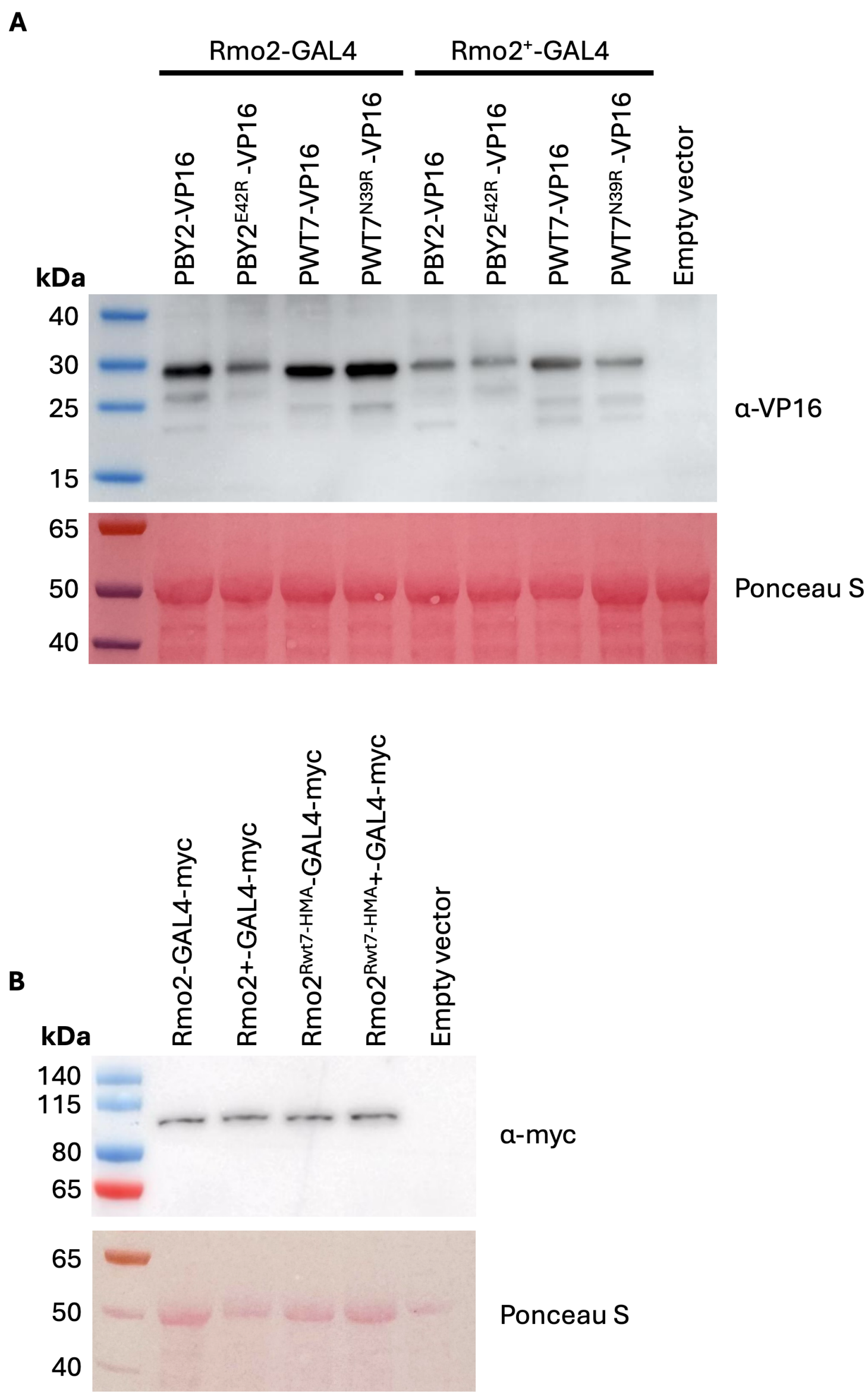

Fig. S7. Confirmation of protein accumulation from the split GAL4 RUBY constructs in *N. benthamiana*. Immunoblot analysis of **(A)** PBY2, PBY2^{E42R}, PWT7, and PWT7^{N39R} (fused to VP16 at their C termini) in *N. benthamiana* leaves co-infiltrated with the respective encoding constructs and a construct encoding Rmo2 or Rmo2+ (fused to the DNA-binding domain of GAL4 at their C termini), and **(B)** Rmo2, Rmo2+, Rmo2$^{Rwt7\text{-}HMA}$, Rmo2$^{Rwt7\text{-}HMA}$+ (fused to GAL4-myc at their C termini). Total proteins were extracted from *N. benthamiana* leaves 4 days after infiltration and separated by SDS-PAGE. The samples were transferred onto a membrane, probed with anti-VP16 or anti-myc antibodies, and signals were detected with a chemiluminescence imager. The membrane was stained with Ponceau S to show equal sample loading.

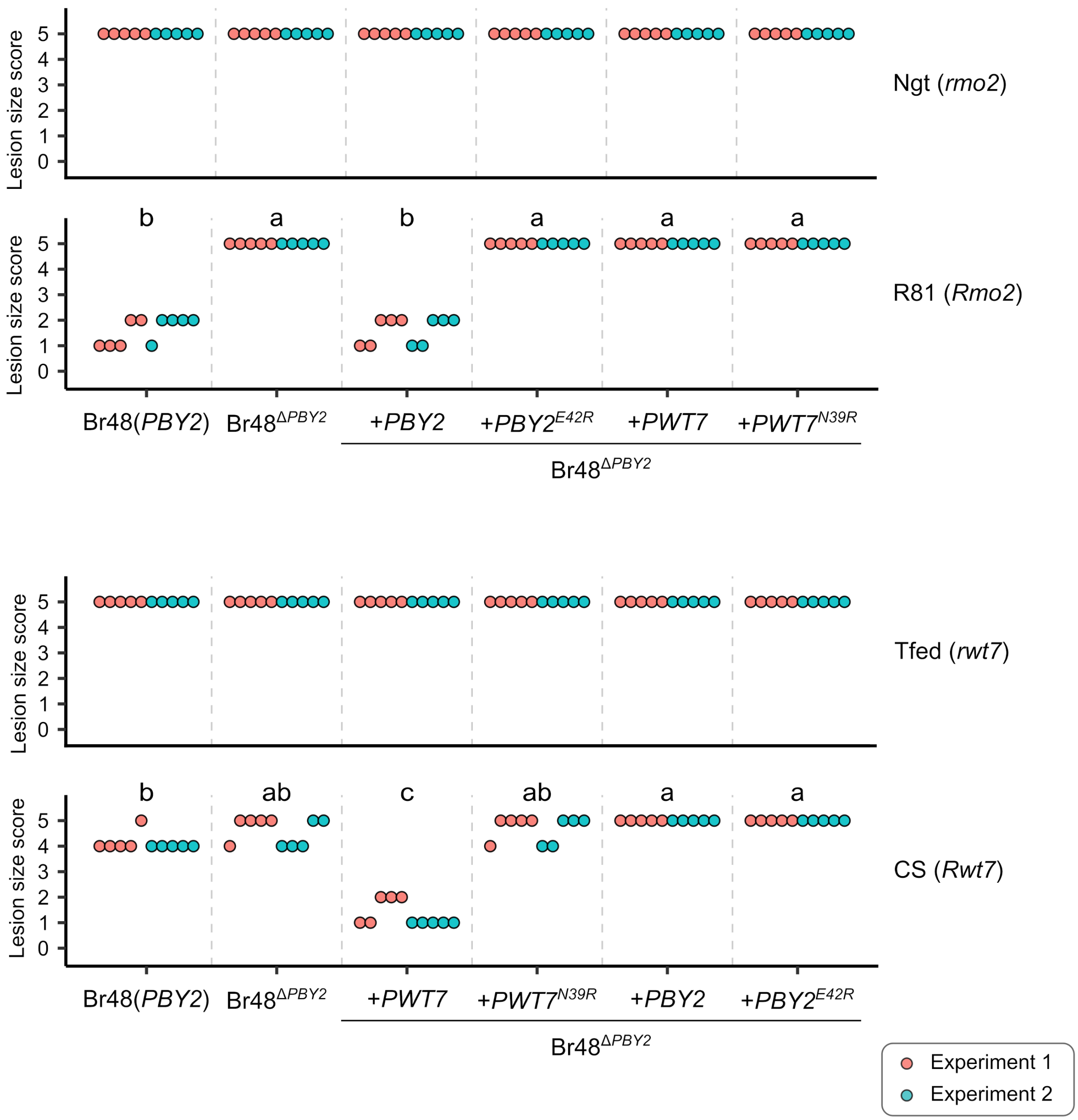

Fig. S8. Lesion size scores of 10 inoculated plants obtained from two independent experiments (five plants per experiment) per fungal strain – barley and wheat genotype combination corresponding to Fig. 2C (A) and Fig. 2D (B). Genotypes of barley and wheat cultivars are indicated in parentheses. Different letters indicate statistically significant differences ($P < 0.05$) determined by Wilcoxon rank sum test with Bonferroni correction. Lesion size scores are rated on six progressive grades as follows: 0, no visible infection; 1, pinhead spots; 2, small lesions (< 1.5 mm); 3, scattered lesions of intermediate size (< 3 mm); 4, large typical lesions; and 5, complete shriveling of leaf blades. Scores 0 to 3 were considered as resistant and 4 to 5 were susceptible.

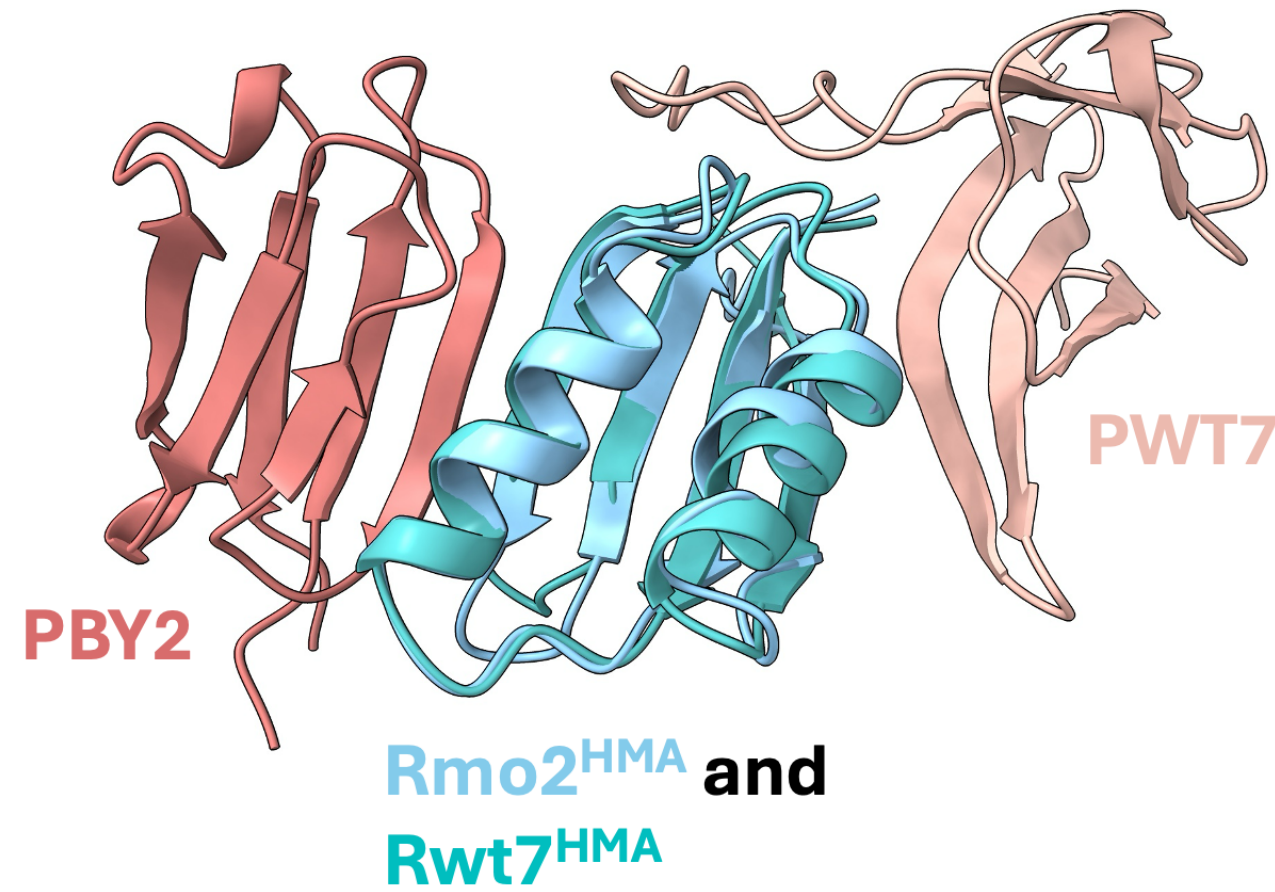

Fig. S9. Structural alignment of the Rmo2^{HMA}/PBY2 and Rwt7^{HMA}/PWT7 complexes. The structures were aligned in ChimeraX.

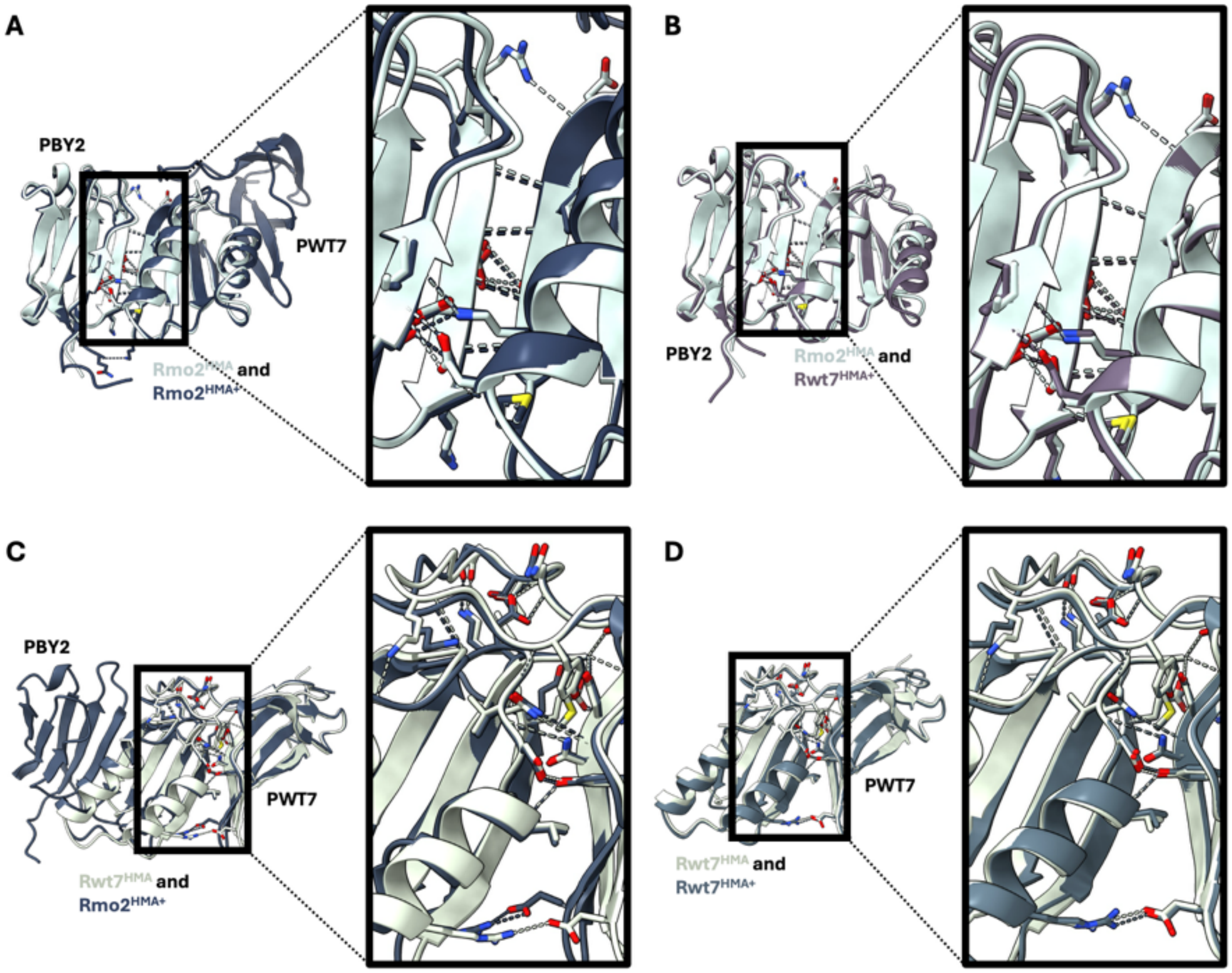

Fig. S10. Engineered gain-of-binding HMA domains have near identical interaction interfaces. (A–D) Structural alignment of Rmo2^{HMA}/PBY2 with **(A)** Rmo2^{HMA+}/PBY2/PWT7 or **(B)** Rwt7^{HMA+}/PBY2, and of Rwt7^{HMA}/PWT7 with **(C)** Rmo2^{HMA+}/PBY2/PWT7 or **(D)** Rwt7^{HMA+}/PWT7. Wild-type structures are shown in light colors and engineered structures are shown in dark colors. Residues involved in hydrogen bonds are shown as sticks. Hydrogen bonds are shown as dotted lines in light or dark colors from the wild-type or engineered structures, respectively. All structures were aligned in ChimeraX.

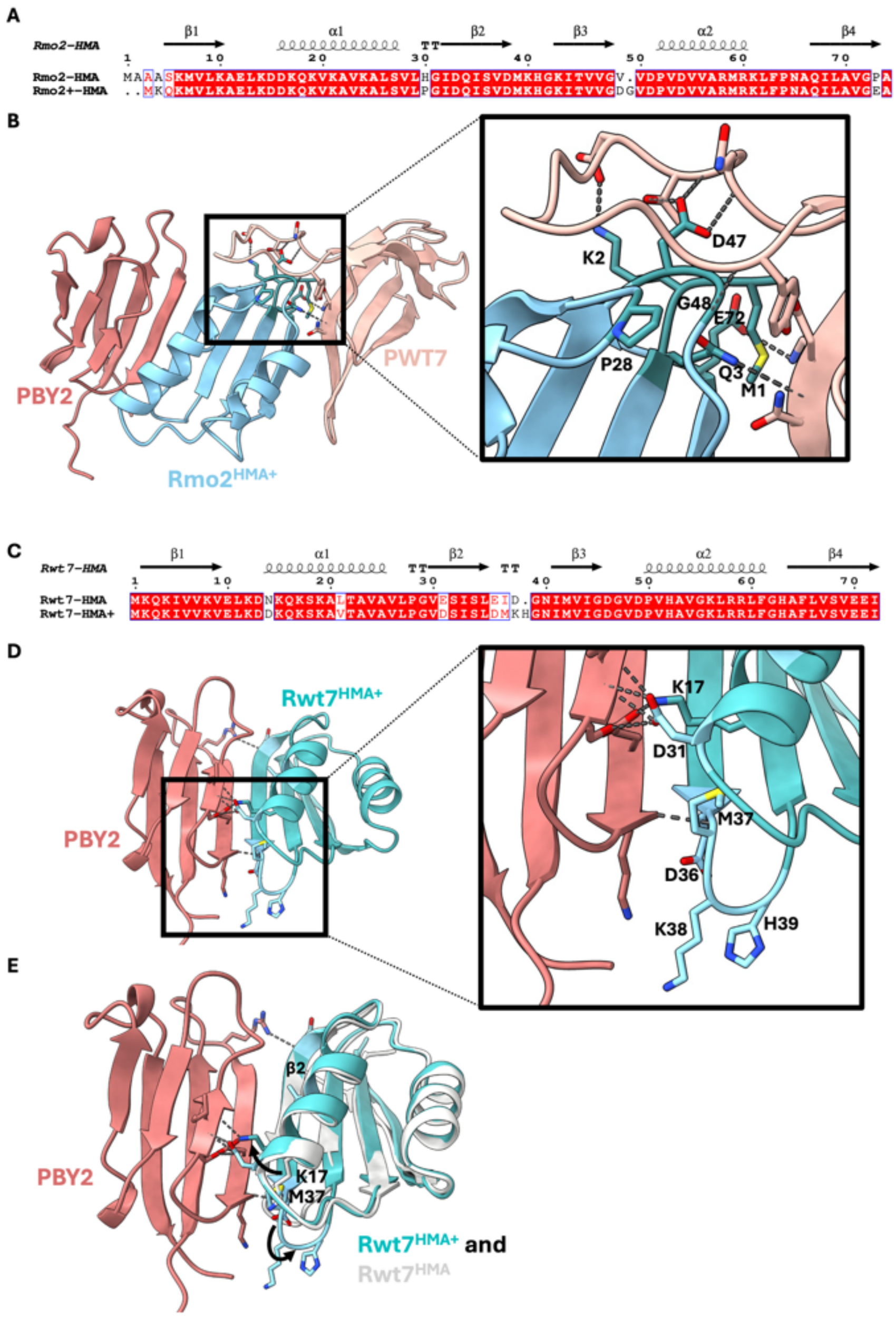

Fig. S11. All residues from the resurfaced HMAs contribute to gain of binding to non-cognate effectors. **(A)** Amino-acid sequence alignment of Rmo2^{HMA} and Rmo2^{HMA+}. **(B)** All mutated residues from Rmo2^{HMA+} and corresponding interactions with PWT7. **(C)** Amino-acid sequence alignment of Rwt7^{HMA} and Rwt7^{HMA+}. **(D)** Mutated residues from Rwt7^{HMA+} and corresponding interactions with PBY2. **(E)** Resurfacing Rwt7^{HMA+} changes its shape, which facilitates its binding to PBY2. The addition of M37 in Rwt7^{HMA+} displaces K17, which normally forms hydrogen bonds with PBY2. The addition of K38 and H39 straightens out β2 to facilitate extensive main chain hydrogen bonds with PBY2. Mutated residues are labeled and shown as sticks. Hydrogen bonds are shown as dotted lines.

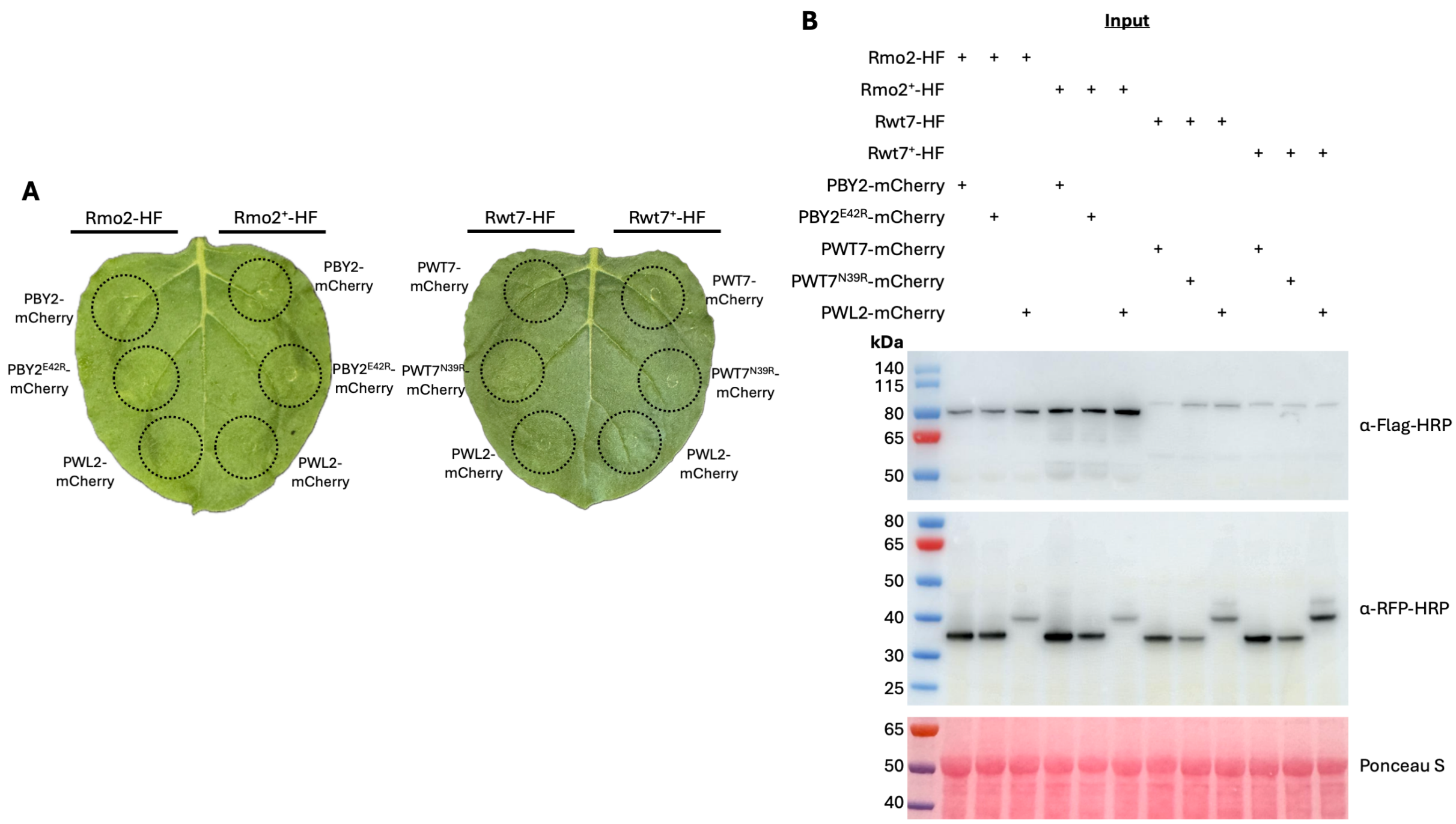

Fig. S12. Wildtype and engineered TKP receptors expressed with corresponding effectors do not cause cell death in *Nicotiana benthamiana*. (A) Co-expression of TKPs with corresponding effectors in *N. benthamiana*. PWL2 was used as a negative control. **(B)** Immunoblot of TKPs and effectors expressed in *N. benthamiana*. Total protein was extracted and separated by SDS-PAGE. The sample was transferred onto a membrane, probed with anti-flag or anti-RFP antibodies and analysed under a chemiluminescence imager. The membrane was stained with Ponceau S to show equal sample loading.

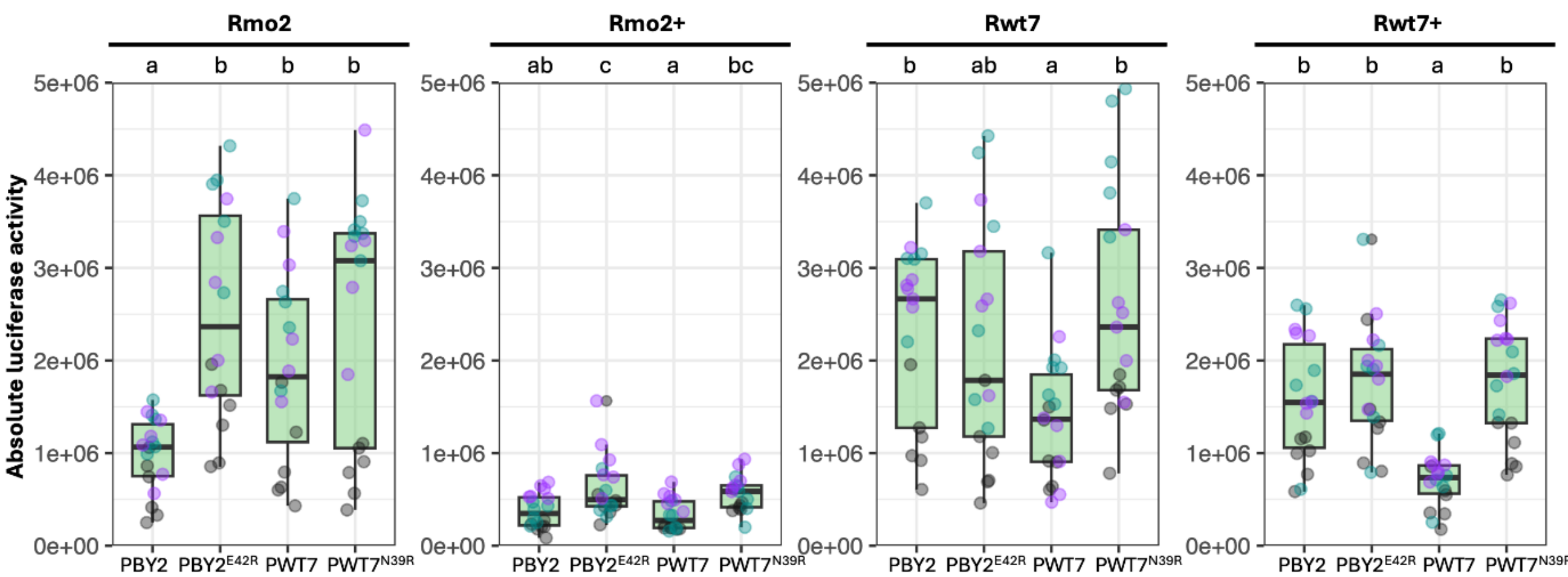

Fig. S13. Lower overall luciferase activity observed with Rmo2+ and Rwt7+ in wheat protoplasts. Absolute luciferase activity in wheat protoplasts (from the Kronos cultivar) transfected with constructs encoding Rmo2, Rmo2+, Rwt7, or Rwt7+ in the presence of the effector PBY2 or PWT7, or the effector variant PBY2^{E42R} or PWT7^{N39R}. Luciferase activity is derived from a *proZmUbi:LUC* reporter. Experiments consisting of six technical replicates were performed three times independently. Different letters indicate significant differences, as determined by one-way ANOVA and *post-hoc* Tukey's honestly significant difference tests at $P < 0.05$.

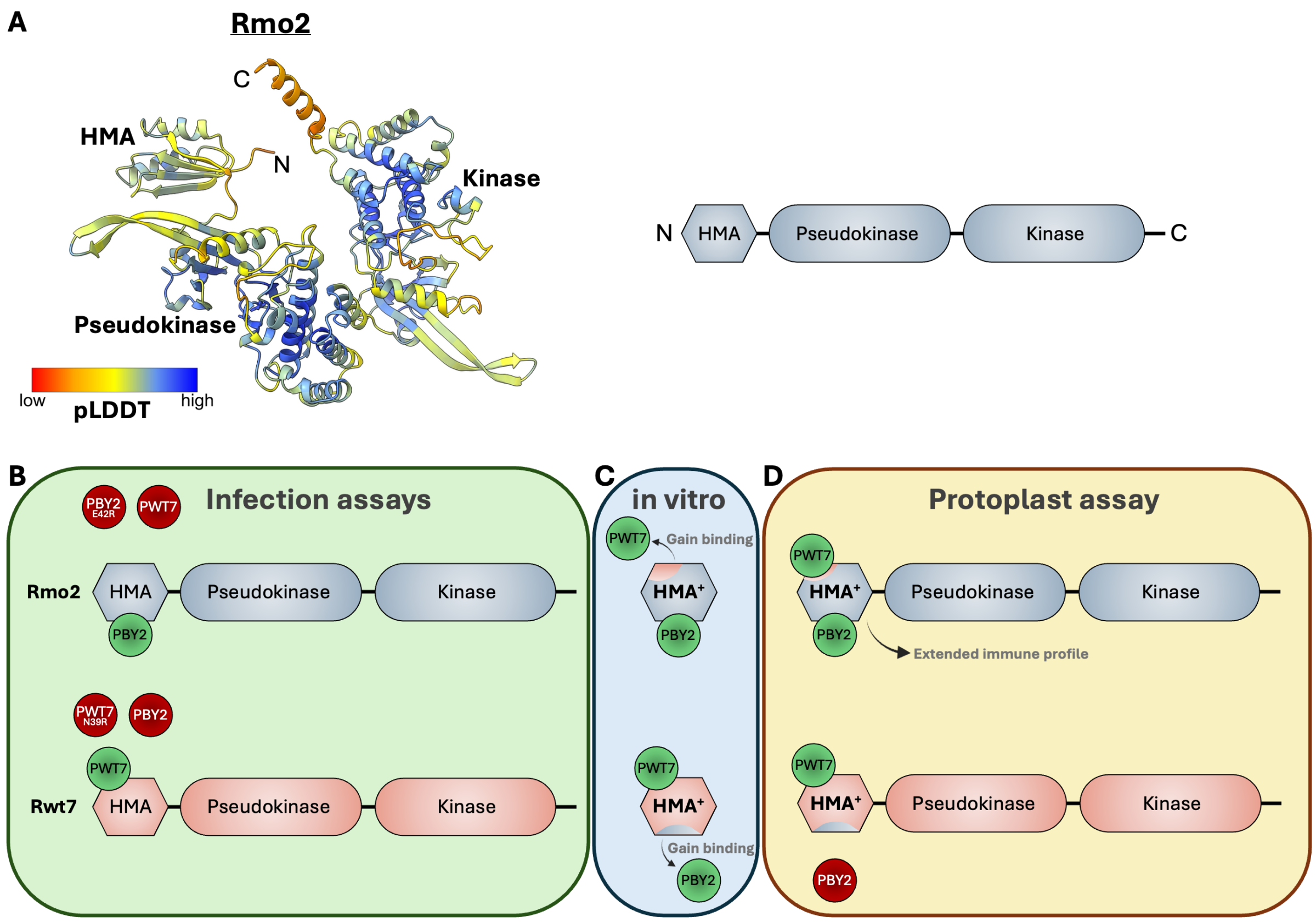

Fig. S14. Schematic of HMA-TKP engineering explored in this manuscript. (A) AlphaFold3 model of Rmo2. The model was coloured by confidence measured by the predicted local distance difference test (pLDDT) on a red to blue rainbow scale. The HMA, pseudokinase and kinase domains are labeled. A simplified schematic of the protein was generated based on the model (right). **(B)** Recognition profiles of Rmo2 (top) and Rwt7 (bottom) determined by infection assays. Effectors depicted as green circles are recognised by Rmo2 or Rwt7 while effectors or effector mutants depicted as red circles are not recognised. **(C)** Engineering of the isolated HMA domains using in vitro assays. The engineered HMA domains (HMA+) were mutated on different surfaces to gain binding of non-corresponding effectors. **(D)** Recognition profiles of the engineered Rmo2+ (top) and Rwt7+ (bottom) in protoplast assays.

Table S1. X-ray data collection, structure solution and refinement statistics for PBY2/Rmo2^{HMA}, PWT7/Rwt7^{HMA}, PBY2/PWT7/Rmo2^{HMA+}, PBY2/Rmo2^{HMA+}, PBY2/Rwt7^{HMA+}, and PWT7/Rwt7^{HMA+}.

	**PBY2/Rmo2^{HMA} (9TFO)**	**PWT7/Rwt7^{HMA} (9TFP)**	**PBY2/PWT7/ Rmo2^{HMA+} (9TFQ)**	**PBY2/Rmo2^{HMA+} (9TFR)**	**PBY2/Rwt7^{HMA+} (9TFS)**	**PWT7/Rwt7^{HMA+} (9TFT)**
Detector	Dectris Eiger2 XE 16M	Dectris Eiger2 XE 16M	Dectris Eiger2 XE 16M	Dectris Eiger2 XE 16M	Dectris Eiger2 XE 16M	Dectris Eiger2 XE 16M
Wavelength (Å)	0.953731	0.953738	0.953727	0.953727	0.953709	0.750006
Space group	P 31 1 2	P 2 21 21	P 1 21 1	P 31 1 2	P 43 21 2	P 1 21 1
Unit cell	89.792 89.792 89.088 90.000 90.000 120.000	39.915 73.609 127.544 90.000 90.000 90.000	56.592 30.590 61.448 90.000 93.015 90.000	90.075 90.075 88.228 90.000 90.000 120.000	105.197 105.197 69.134 90.000 90.000 90.000	40.524 66.174 65.747 90.000 104.851 90.000
Average mosaicity (°) [b]	0.00	0.00	0.00	0.00	0.00	0.00
Resolution (Å)	89.09–2.20 (2.27–2.20)	63.85–1.60 (1.63–1.60)	61.36–1.40 (1.42–1.40)	88.23–2.60 (2.72–2.60)	74.39–1.80 (1.84–1.80)	66.17–0.99 (1.01–0.99)
Total no. of reflections	438,495 (38,796)	1,364,358 (67,667)	566,095 (27,819)	531,069 (66,239)	1,680,137 (96,874)	2,555,845 (125,512)
No. of unique reflections	21,089 (1,838)	50,590 (2,424)	41,916 (2,032)	12,793 (1,545)	36,570 (2,113)	186,021 (9,174)
Completeness (%)	100.0 (100.0)	100.0 (100.0)	100.0 (100.0)	100.0 (100.0)	100.0 (100.0)	100.0 (100.0)
Multiplicity	20.8 (21.1)	27.0 (27.9)	13.5 (13.7)	41.5 (42.9)	45.9 (45.8)	13.7 (13.7)
Mean I /s (I)	26.6 (1.7)	32.7 (2.0)	19.8 (1.8)	24.7 (2.0)	22.2 (3.9)	15.2 (1.5)
Rmerge	0.060 (0.931)	0.052 (1.025)	0.054 (1.287)	0.106 (2.412)	0.145 (1.227)	0.062 (1.610)
Rmeas [c]	0.063 (0.977)	0.054 (1.063)	0.058 (1.386)	0.108 (2.469)	0.148 (1.254)	0.066 (1.740)
Rpim [d]	0.019 (0.295)	0.014 (0.280)	0.022 (0.514)	0.023 (0.526)	0.030 (0.256)	0.025 (0.657)
$CC_{1/2}$ [b]	1.000 (0.944)	1.000 (0.961)	1.000 (0.872)	1.000 (0.888)	1.000 (0.971)	1.000 (0.817)
Matthews coeff. ($Å^3$ Da^{-1}) [e]	2.16	2.67	2.10	3.34	3.21	2.46
Resolution range (Å)	77.88–2.20	63.85–1.60	61.36–1.40	78.13–2.60	74.50 –1.80	63.63–0.99
R_{work} (%) [g]	0.213	0.161	0.154	0.188	0.173	0.140
R_{free} (%) [h]	0.267	0.198	0.193	0.262	0.210	0.153
No. of non-H atoms						
Amino acids	2112	2413	1737	2056	2144	2503
Ligand	9	64	12	0	89	0
Water	65	287	194	26	305	477
Average B-factor ($Å^2$)	75.581	37.751	26.850	93.148	26.834	14.631
RMSD from ideal geometry						
Bond lengths (Å)	0.0071	0.0103	0.0107	0.0130	0.0112	0.0125
Bond angles (°)	1.796	1.776	1.929	2.772	1.893	1.948
Ramachandran plot, residues in (%)[i]						
Favored regions	95.13	99.00	99.05	93.85	99.25	98.34
Allowed regions	3.75	1.00	0.95	5.77	0.75	1.66
Outlier regions	1.12	0.00	0.00	0.38	0.00	0.00

[a] The values in parentheses are for the highest-resolution shell.
[b] Calculated with AIMLESS.
[c] Rmeas = Σhkl (N(hkl) / [N(hkl) – 1])^(1/2) Σi | Ii(hkl) – (1 / N(hkl)) Σj Ij(hkl) | / Σhkl Σi Ii(hkl), where Ii(hkl) is the intensity of the *i*th measurement of an equivalent reflection with indices hkl.
[d] Rpim = Σhkl (1 / [N(hkl) – 1])^(1/2) Σi | Ii(hkl) – (1 / N(hkl)) Σj Ij(hkl) | / Σhkl Σi Ii(hkl).
[e] Calculated with MATTHEWS_COEF within the CCP4 suite.
[f] Generated by Crank pipeline in the CCP4 suite.
[g] Rwork = Σall | Fobs – Fcalc | / Σall | Fobs |, where Fobs and Fcalc are the observed and calculated structure factor amplitudes.
[h] Rfree is equivalent to Rwork but calculated with reflections (5%) omitted from the refinement process.
[i] Calculated with MolProbity.

Table S2. Constructs used in this study

Construct	Domain boundary	Backbone	Purpose
GB1-PBY2	PBY2$^{21–85}$	pPGN-C	*E. coli* production, analytical SEC, ITC, structures
GB1-PWT7	PWT7$^{22–98}$	pPGN-C	*E. coli* production, analytical SEC, ITC, structures
GB1-Rmo2^{HMA}	Rmo2$^{1–77}$	pPGN-C	*E. coli* production, analytical SEC, ITC
GB1-Rwt7^{HMA}	Rwt7$^{1–77}$	pPGN-C	*E. coli* production, analytical SEC
Rmo2^{HMA}	Rmo2$^{1–77}$	pPGC-K	*E. coli* production, structures
Rwt7^{HMA}	Rwt7$^{1–77}$	pPGC-K	*E. coli* production, structures
GB1-PBY2^{E42R}	PBY2$^{21–85}$	pPGN-C	*E. coli* production, analytical SEC, ITC
GB1-PWT7^{N39R}	PWT7$^{22–98}$	pPGN-C	*E. coli* production, analytical SEC, ITC
Rwt7-6His	Rwt7$^{1–77}$	pPGC-C	*E. coli* production, ITC
Rmo2^{HMA+}-6His	Rmo2$^{1–77}$	pPGC-C	*E. coli* production, ITC
Rwt7^{HMA+}-6His	Rwt7$^{1–77}$	pPGC-C	*E. coli* production, ITC
Rmo2^{HMA+}	Rmo2$^{1–77}$	pPGC-K	*E. coli* production, structures
Rwt7^{HMA+}	Rwt7$^{1–77}$	pPGC-K	*E. coli* production, structures
35S:Rmo2-GAL4:T35S	Rmo2$^{1–692}$	pICSL22027	RUBY assay
35S:Rwt7-GAL4:T35S	Rwt7$^{1–758}$	pICSL22027	RUBY assay
35S:Rmo2$^{Rwt7\text{-}HMA}$-GAL4:T35S	Rwt7$^{1–72}$; Rmo2$^{75–692}$	pICSL22027	RUBY assay
35S:Rmo2+-GAL4:T35S	Rmo2$^{1–692}$	pICSL22027	RUBY assay
35S:Rmo2$^{Rwt7+\text{-}HMA}$-GAL4:T35S	Rwt7$^{1–72}$; Rmo2$^{75–692}$	pICSL22027	RUBY assay
35S:PBY2-VP16:T35S	PBY2$^{21–85}$	pICSL22026	RUBY assay
35S:PBY2^{E42R}-VP16:T35S	PBY2$^{21–85}$	pICSL22026	RUBY assay
35S:PWT7-VP16:T35S	PWT7$^{22–98}$	pICSL22026	RUBY assay
35S:PWT7^{N39R}-VP16:T35S	PWT7$^{22–98}$	pICSL22026	RUBY assay
35S:Rmo2-GAL4-myc:T35S	Rmo2$^{1–692}$	pICSL22027	Immunoblotting
35S:Rmo2$^{Rwt7\text{-}HMA}$-GAL4-myc:T35S	Rwt7$^{1–72}$; Rmo2$^{75–692}$	pICSL22027	Immunoblotting

35S:Rmo2+-GAL4-myc:T35S	$Rmo2^{1–692}$	pICSL22027	Immunoblotting
35S:$Rmo2^{Rwt7+-HMA}$-GAL4-myc:T35S	$Rwt7^{1–72}$; $Rmo2^{75–692}$	pICSL22027	Immunoblotting
pZH2Bik-PWT7	$PWT7^{22–98}$	pZH2Bik	Protoplast assay
pZH2Bik-$PWT7^{N39R}$	$PWT7^{22–98}$	pZH2Bik	Protoplast assay
pZH2Bik-Rmo2	$Rmo2^{1–692}$	pZH2Bik	Protoplast assay
pZH2Bik-Rmo2+	$Rmo2^{1–692}$	pZH2Bik	Protoplast assay
pZH2Bik-Rwt7	$Rwt7^{1–758}$	pZH2Bik	Protoplast assay
pZH2Bik-Rwt7+	$Rwt7^{1–758}$	pZH2Bik	Protoplast assay
pZH2Bik-PBY2-myc	$PBY2^{21–85}$	pZH2Bik	Immunoblotting
pZH2Bik-$PBY2^{E42R}$-myc	$PBY2^{21–85}$	pZH2Bik	Immunoblotting
pZH2Bik-PWT7-myc	$PWT7^{22–98}$	pZH2Bik	Immunoblotting
pZH2Bik-$PWT7^{N39R}$-myc	$PWT7^{22–98}$	pZH2Bik	Immunoblotting
